# Polygenic sex determination in the cichlid fish *Astatotilapia burtoni*

**DOI:** 10.1186/s12864-016-3177-1

**Published:** 2016-10-26

**Authors:** Natalie B. Roberts, Scott A. Juntti, Kaitlin P. Coyle, Bethany L. Dumont, M. Kaitlyn Stanley, Allyson Q. Ryan, Russell D. Fernald, Reade B. Roberts

**Affiliations:** 1 Department of Biological Sciences and W. M. Keck Center for Behavioral Biology, North Carolina State University, Raleigh, NC USA; 2 Department of Biology, Stanford University, Stanford, CA USA

**Keywords:** Sex determination, Polygenic sex determination, Cichlid, Fish, Evolution, Astatotilapia burtoni

## Abstract

**Background:**

The East African riverine cichlid species *Astatotilapia burtoni* serves as an important laboratory model for sexually dimorphic physiology and behavior, and also serves as an outgroup species for the explosive adaptive radiations of cichlid species in Lake Malawi and Lake Victoria. An astounding diversity of genetic sex determination systems have been revealed within the adaptive radiation of East African cichlids thus far, including polygenic sex determination systems involving the epistatic interaction of multiple, independently segregating sex determination alleles. However, sex determination has remained unmapped in *A. burtoni.* Here we present mapping results supporting the presence of multiple, novel sex determination alleles, and thus the presence of polygenic sex determination in *A. burtoni*.

**Results:**

Using mapping in small families in conjunction with restriction-site associated DNA sequencing strategies, we identify associations with sex at loci on linkage group 13 and linkage group 5–14. Inheritance patterns support an XY sex determination system on linkage group 5–14 (a chromosome fusion relative to other cichlids studied), and an XYW system on linkage group 13, and these associations are replicated in multiple families. Additionally, combining our genetic data with comparative genomic analysis identifies another fusion that is unassociated with sex, with linkage group 8–24 and linkage group 16–21 fused in *A. burtoni* relative to other East African cichlid species.

**Conclusions:**

We identify genetic signals supporting the presence of three previously unidentified sex determination alleles at two loci in the species *A. burtoni*, strongly supporting the presence of polygenic sex determination system in the species. These results provide a foundation for future mapping of multiple sex determination genes and their interactions. A better understanding of sex determination in *A. burtoni* provides important context for their use in behavioral studies, as well as studies of the evolution of genetic sex determination and sexual conflicts in East African cichlids.

**Electronic supplementary material:**

The online version of this article (doi:10.1186/s12864-016-3177-1) contains supplementary material, which is available to authorized users.

## Background

Sex determination is absolutely fundamental to the fitness of sexually reproducing organisms, yet the “switches” modulating female versus male development are incredibly diverse [1]. This diversity arises from surprisingly numerous transitions in sex determination mechanisms across taxa, yet little is know about the evolutionary and developmental forces underlying these transitions. Given the current catalog of identified sex determination mechanisms, fish are arguably the taxon with the highest diversity of sex determination mechanisms [2]. Broadly, sex determination mechanisms are categorized as environmental (e.g., temperature during development), behavioral (e.g., social status), or genetic (e.g., the *Sry* gene in mammals). Historically, the architecture of genetic sex determination was considered relatively simple, with a single inherited cue driving both primary gonadal sexual development, and producing resounding secondary sexual effects throughout the organism [3]. In vertebrates with genetic sex determination, sex determination alleles act as this cue. In species with XY sex determination systems, inheritance of the Y allele is associated with male development (XX females, XY males); with ZW systems the W allele is associated with female development (ZW females, ZZ males). When a sex determination allele arises on an autosome, the evolutionary trajectory of that chromosome can change, with new selection pressures potentially leading to local recombination suppression and heteromorphy over time [4]. Thus sex determination alleles can exist on highly degenerated sex chromosomes such as the Y chromosome in mammals [5], within defined sex loci with local recombination suppression [6], or as a simple polymorphism with no associated chromosomal changes [7].

As sex determination alleles are cataloged, surprising overlap suggests repeated modification of a few pathways across broad taxa to modulate sexual development. Specifically, members of the *Sry-related HMG box* (*sox*) and *doublesex and mab-3 related* (*dmrt*) families of transcription factors, and anti-Müllerian hormone (*amh*) pathway members, have repeatedly evolved as sex determination factors (reviewed in [8]; see also [9, 10]). Studies in fish have been key to identifying the diversity of vertebrate genetic sex determiners, with a role for *dmrt* first identified in medaka, and later demonstrated in chicken and frog (*Xenopus*) [6, 11, 12]; similarly the role of the *amh* pathway was first identified in pejerrey and fugu, and later in platypus [7, 13, 14]. Moreover, recent identification of sex determiners such as *gsdf* and *sexually dimorphic on the Y-chromosome* (*sdY*) within fish make it clear that sex determination mechanisms are not limited to the above pathways [15, 16].

It was also within fish that polygenic sex determination was discovered [17, 18], demonstrating that genetic sex determination need not be monogenic in nature. In polygenic sex determination systems, multiple, independently segregating sex determination alleles interact to determine sex within a species [3]. Historically, examples of polygenic sex determination were considered anomalous or evolutionarily transitory; however, with broader surveys and readily available genomic tools, polygenic sex determination has been identified in multiple animal taxa, including fish, mammals, insects, and copepods [3, 19], suggesting that polygenic sex determination is a common and stable evolutionary strategy.

In fish, polygenic sex determination systems exist in both single- and multi-locus configurations. In platyfish (*Xiphophorus maculatus*), three alleles (X, Y, and W) segregate at the sex determination locus, interacting to determine male versus female development in what is sometimes referred to as an XYW system [18, 20]. In some East African cichlids (e.g., *Metriaclima* spp. from Lake Malawi), XY and ZW sex determination systems reside on distinct chromosomes, and epistasis between genotypes at the two loci determines male versus female development [21, 22]. Polygenic sex determination in fish can involve more than two sex determination alleles. For example, mapping sex as a quantitative trait in a hybrid cross of two cichlid species identified at least five different loci interacting in an epistatic hierarchy to determine sex [23]. In sea bass, the genetic architecture of sex determination may be equally polygenic, with recent mapping efforts identifying at least four loci in the genome associated with sex determination [24].

In zebrafish (*Danio rerio*), several independent studies indicated a highly polygenic basis of sex determination in standard laboratory strains [25–27]; however, a more recent study revealed a monogenic ZW sex determination system in natural populations, with the W allele absent from laboratory strains, presumably lost during domestication [28]. The zebrafish findings serve as a cautionary tale for interpreting laboratory results without natural context, but also speak to the lability of sex determination and development in fishes. Such lability has been known for some time, with the loss of a natural sex chromosome and arisal of a novel autosomal sex determiner during selective breeding in guppies (*Poecilia reticulata*) described nearly a century ago [29]. In natural context, the forces underlying such transitions between sex determination systems are poorly understood.

The adaptive radiation of African cichlid fish is a powerful model system for the study of evolution and development, and an astounding diversity of genetic sex determination loci have been revealed from just a handful of studied species [22, 23, 30–35]. These sex determination loci exist in various contexts, including polygenic sex determination [22, 23, 30, 31], on supernumerary chromosomes [32], and in regions of potential recombination suppression indicative of early sex chromosome evolution [21, 36, 37]. The riverine cichlid *Astatotilapia burtoni* has served as a longstanding model of sexual physiology, behavior, and related gene expression [38], yet until recently sex determination in the species was largely unexamined. A recent study described sex-specific transcriptional differences in the gonad of *A. burtoni* [39], and another inferred the presence of an XY system in the species using crossing experiments with hormonally sex-reversed individuals [40]. However, direct genetic mapping of sex determination was not previously performed for *A. burtoni*.

Here, we describe use of double digest restriction associated DNA sequencing (ddRADseq) to perform genome scans for sex-associated loci in small *A. burtoni* families, followed by replication in additional, independent families. We order the *A. burtoni* genome scaffolds to two other, higher quality cichlid genomes to orient mapping. Mapping results indicate the presence of an XYW locus on linkage group 13 (LG13), and an XY locus on LG5-14 (a chromosome fusion relative to other cichlids). The evidence for at least three sex determination alleles strongly supports the presence of polygenic sex determination in *A. burtoni*, though we discuss caveats related to sex reversal and lability of sex determination during fish domestication. Additionally, haplotype data define an additional pair of linkage groups involved in the other *A. burtoni-*specific chromosome fusion.

## Results and discussion

### Comparative scaffolding of the *A. burtoni* genome

*A. burtoni* was one of five cichlid species sequenced as part of the cichlid genome project, but was also the species with the most fragmented genome assembly (scaffold N50 of 1.2 Mb, versus 2.5–4.4 Mb for the other four species; [41]). In order to provide chromosome-level organization of *A. burtoni* scaffolds for mapping, we ordered and oriented them to anchored genomes of two of other cichlid species (Additional files 1 and 2). The Nile tilapia (*Oreochromis niloticus*) genome assembly was anchored to a high-resolution radiation hybrid map, providing a golden path [41, 42]. Genome scaffolds of *Metriaclima zebra*, a Lake Malawi species, were also anchored to a RADseq-based genetic map [43]. *A. burtoni* scaffold assignment was consistent by linkage group for both species, but regular inconsistencies in scaffold order indicate a moderate level of intrachromosomal rearrangement between *O. niloticus* and *M. zebra.* We use the *O. niloticus* scaffolding for the following analysis because it provided the most parsimonious ordering based on our marker data, including producing continuous, uninterrupted mapping intervals. We number *A. burtoni* linkage groups based on synteny with established *O. niloticus* linkage group numbering, per convention in the cichlid genetics community [42].

### Identification of two sex determination loci

**Fig. 1:**
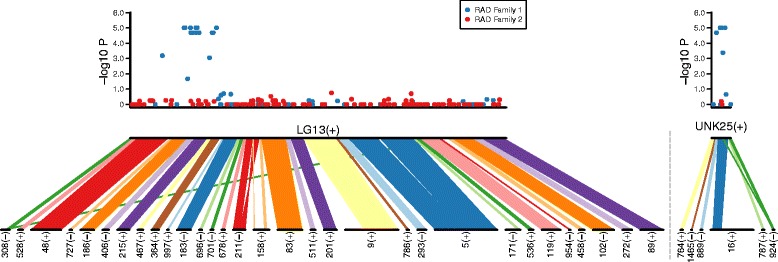
Association of sex with SNP markers on LG13 in family 1. Association plotted as the negative log of Fisher’s exact test *p* value of marker alleles with sex (*top*), aligned to *A. burtoni* genome scaffolds ordered and oriented to LG13 and UNK25 of the anchored *O. niloticus* genome assembly (*bottom*). Associated scaffolds aligning to *O. niloticus* UNK25 (*right*) should likely be placed on LG13, in or adjacent to the peak of association with sex

Two *A. burtoni* families with near-equal sex ratios were genotyped using a ddRADseq strategy, and resulting SNP marker genotypes were tested for association with gonadal sex (Table 1). Markers with significant *p* values (*p* < 0.01) were further sorted by allele frequency in each sex, to search for minor alleles at intermediate frequency in one sex, and absent in the other, following a dominant model of sex determination (Additional file 3). Both families had numerous markers with significant associations with phenotypic sex, and alignment of marker sequences to the tilapia genome revealed clustering of the associated markers by linkage group. In one family, marker genotypes in a region of LG13 show near perfect association with sex, as do markers on unanchored portions of the tilapia assembly including UNK25 and UNK107 (Fig. 1, Additional file 3). Based on association and haplotype patterns we predict that sex-associated scaffolds aligning to UNK25 and UNK107 should be placed on LG13, within or adjacent to the sex associated scaffolds aligning there. Because maternal alleles at LG13 co-segregate with sex, we designate this family as having sex determined by a ZW system.

**Table 1:** Summary of ddRADseq mapping families

Family	M	F	Reads^a^	Stacks^a^	Polymorphic stacks^b^	Lowest *p*-value^c^	Corresponding Tilapia linkage groups^d^
RAD 1	12	12	9.7 × 10^6^	435,006	4,777	9.6 × 10^−6^	LG13, UNK25, UNK107
RAD 2	9	8	5.9 × 10^6^	341,384	4,268	1.4 × 10^−3^	LG5, LG14

Families with number of male (M) and female (F) offspring. ^a^Average reads and stacks per offspring. ^b^Total number of polymorphic stacks used for association mapping, requiring a minimum stack depth of 10 to call homozygotes, and genotypes called for 20 of 24 offspring for RAD 1, and 15 of 17 offspring for RAD 2. ^c^The lowest *p*-value obtained for Fisher’s exact test of allele association with sex, shared by multiple markers (Additional file 3). ^d^Linkage groups with markers with lowest *p*-value association

**Fig. 2:**
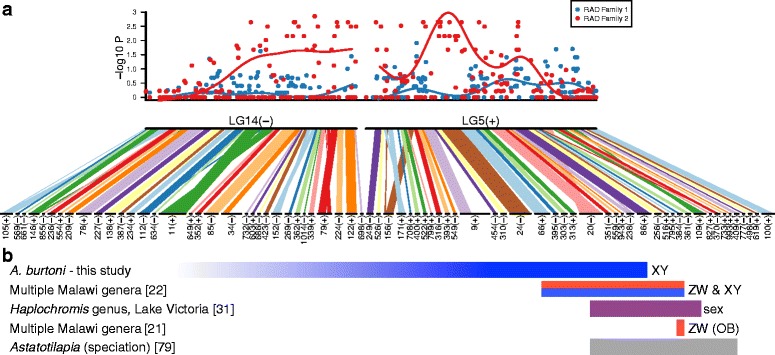
Association of sex with SNP markers on LG5-14 in family 2, with comparisons to other mapping studies. **a** Association plotted as the negative log of Fisher’s exact test *p* value of marker alleles with sex (*top*), aligned to *A. burtoni* genome scaffolds ordered and oriented to LG5 and LG14 of the *O. niloticus* genome assembly (*bottom*). In *A. burtoni*, LG5 and LG14 are fused relative to other cichlids. *Lines* on association plot indicate sliding window analysis of association data (window, 10 SNPs; step, 1 SNP). **b** Comparison of sex-associated markers with previous mapping studies in cichlids, with numbers linking to references. *Colored bars* indicate range of sex-associated markers relative to *A. burtoni* scaffolds above in **a**. Kudo et al. [31] described association with sex without indicating an XY or ZW system. Roberts et al. [21] mapped the orange blotch (OB) pigmentation allele, tightly linked to a W sex determination allele

In a second family, associated RAD markers consistently aligned to tilapia LG5 and LG14 (Fig. 2a, Additional file 3). Previous karyotype analysis demonstrated that *A. burtoni* has two fused chromosome pairs relative to all other haplochromine cichlids surveyed [44]. Examination of our associated marker genotypes at LG5 and LG14 revealed clear haplotypes with no recombinants spanning the two linkage groups (Additional file 4), demonstrating that LG5 and LG14 constitute one of the chromosome fusions in the *A. burtoni* karyotype relative to tilapia; we refer to this fusion as LG5-14 in *A. burtoni* (Fig. 2). Paternal alleles co-segregate with sex at LG5-14, and thus we designate it as carrying an XY sex determination system.

Because the two discovery families were relatively small and provided relatively low power to detect associations, we confirmed association of these loci with phenotypic sex by testing 25 additional families with simple sequence repeat (SSR) markers on LG13 and LG5-14 (Table 2, Additional files 5 and 6). Segregation of parental alleles at either LG13 or LG5-14 is associated with sex determination in the majority of families tested. In families where genotypes at LG5-14 are associated with sex, the sire’s alleles are consistently associated with sex of offspring (Table 2), replicating findings from the discovery family, and providing strong evidence for an XY sex determination system on LG5-14. Where multiple families from the same LG5-14 XY sire were tested, the same paternal haplotype is consistently associated with male sex in offspring (Additional file 6). We infer that this paternal haplotype is tightly linked to a dominant Y male sex determination allele.

**Table 2:** Association of genetic markers with sex in families

Family	Sire	Dam	M	F	M:F	LG5-14 Y *p*	LG13 Y *p*	LG13 W *p*	System
RAD 1	1	1	12	12	0.50	0.6843	NI	**0.0001**	LG13 ZW
RAD 2	2	2	9	8	0.53	**0.0023**	0.6199	1	LG5-14 XY
1	1	1	10	36	0.22	0.1639	NI	**0.0012**	LG13 ZW
2	3	3	8	8	0.50	**0.0087**	NI	**0.0001**	LG13 ZW
3	3	3	9	8	0.53	0.3469	NI	**0.0152**	LG13 ZW
4	4	4	6	26	0.19	NI	NI	**0.0192**	LG13 ZW
5	5	5	4	6	0.40	1	0.5714	**0.0048**	LG13 ZW
6	6	6	4	10	0.29	0.2208	1	**0.015**	LG13 ZW
7	6	7	10	9	0.53	0.1534	NI	**0.009**	LG13 ZW
8	7	8	2	13	0.13	1	**0.0128**	NI	LG13 XY
9	7	9	16	22	0.42	0.72	**0.0001**	0.752	LG13 XY
10	8	10	12	14	0.46	**0.0001**	0.2177	1	LG5-14 XY
11	8	11	12	10	0.55	**0.003**	0.0092	0.6914	LG5-14 XY
12	8	12	15	11	0.58	**0.0001**	0.4283	0.4279	LG5-14 XY
13	8	13	12	7	0.63	**0.0002**	0.3261	0.3498	LG5-14 XY
14	8	14	4	2	0.67	0.0667	0.4	1	LG5-14 XY
15	9	15	12	10	0.55	**0.0015**	0.3246	0.1312	LG5-14 XY
16	9	16	13	8	0.62	**0.0237**	NI	0.2031	LG5-14 XY
17	9	17	15	8	0.65	**0.0083**	0.2213	NI	LG5-14 XY
18	10	18	11	5	0.69	**0.0256**	0.5962	1	LG5-14 XY
19	11	19	5	4	0.56	0.444	**0.0079**	NI	LG13 XY + LG5-14 XY
20	12	20	9	11	0.45	1	1	0.1789	Unknown
21	6	21	12	26	0.32	0.6792	0.2305	NI	Unknown
22	13	22	7	9	0.44	0.3024	0.1262	0.3147	Unknown
23	13	23	14	17	0.45	1	1	0.4809	Unknown
24	13	24	20	23	0.47	0.3652	0.7619	0.0755	Unknown
25	13	25	22	20	0.52	0.3783	**0.0414**	1	Unknown

Families including identification number of sire and dam, number of male (M) and female (F) offspring, and sex ratio (M:F). *p* values are from Fisher’s exact test of association of SSR marker data with phenotypic sex, testing the sire’s alleles for Y associations, and dam’s alleles for W association, with bold values indicating *p* < 0.05, and NI indicating markers tested were not informative. System indicates inferred sex determination system in family from marker data within and among families. An expanded version of this table with markers used, and a table with marker genotype data is available in Additional files 5 and 6.

In families where LG13 is associated with sex determination, either a maternal or a paternal allele can be found associated with sex, depending on the family tested (Table 2). In several families, a maternal haplotype segregates with sex in the offspring, and the same maternal haplotype is associated with female sex in multiple families, supporting the presence of a dominant W female sex determination allele (Additional file 6). Surprisingly, paternal alleles at LG13 co-segregate with sex of offspring in families from two sires, suggesting that a dominant Y male sex determination allele may also be present on LG13 (Table 2, Additional file 6). Differences in parental inheritance of sex determination alleles among families with LG13 sex determination suggest that an XYW sex determination system akin to that found in platyfish may be present at LG13; however, occasional sex reversal could also account for observed patterns in the absence of a LG13 Y allele (see below).

Several families showed no association of sex with markers at LG13 or LG5-14 (Table 2). These families allow the possibility of additional, unidentified sex determination loci in *A. burtoni*. Alternatively, interactions among identified sex determination alleles, or between sex determination alleles and the environment, could confound interpretation.

### Interactions between sex loci

Because we find multiple loci and alleles that appear to impact sex, we would expect some families to be segregating multiple alleles that would interact to influence sex determination. Studies in both tilapias and Lake Malawi cichlids with polygenic sex determination have revealed clear patterns of epistasis relating to phenotypic sex [21–23, 30]. Unfortunately, we find no clear examples of such interactions in our families. One small family (Family 19) has perfect segregation of sex with paternal alleles at LG13, supporting the sire having an LG13 XY genotype. Two sons but no daughters in the family also inherit a multi-marker haplotype at LG5-14 associated with the LG5-14 Y in other families (Additional file 7). We hypothesize that the genotype of the sire and two sons in this family is LG5-14 XY, LG13 XY, and thus that individuals with a Y allele at each locus are viable and develop as males; however, this interpretation is based on a single, small family and is thus very tentative.

In families tested where sex is not clearly associated with alleles at either LG13 or LG5-14 alone (Table 2), inter- and intra-locus interactions may be present that do not conform to our expected patterns of allelic dominance. Additionally, some families were not included in this study because they had extreme sex ratios (sometimes single-sex), and/or few offspring, preventing meaningful interpretation of inheritance patterns; however, interaction of multiple sex alleles may produce skewed sex ratios or non-viable genotypes. In cichlids and other fish species, co-segregation of both Y and W alleles in families produces female-biased sex ratios [18, 22, 30], and in pygmy mice with an XYW system, mating of some sex genotypes results in inviability of a portion of their offspring [45]. Parnell and Streelman described five distinct sex determination loci (three XY and two ZW systems) in a Lake Malawi cichlid mapping cross, demonstrating that sex ratios represent the outcome of complex interactions between relatively strong and weak sex determination alleles [23]. Thus, epistasis may be difficult to characterize, particularly in small families, and further work is needed to understand the phenotypic outcomes of interactions between the sex determination alleles identified.

### Sex ratios and penetrance

**Fig. 3:**
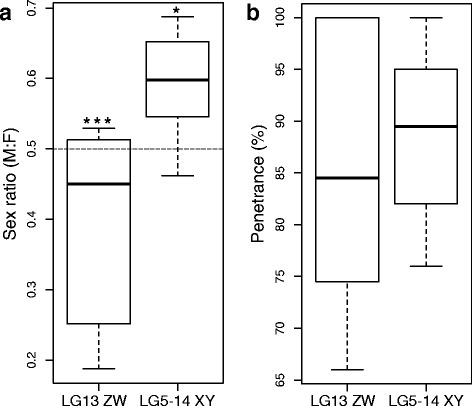
Sex ratios and penetrance by sex determination system. **a** Sex ratio by sex determination system. *Dashed line* indicates a 1:1 sex ratio, with values lower than the *line* indicating a female-biased sex ratio, and those above the line a male-biased sex ratio. (LG13 ZW, *n* = 8 families, 63 males and 115 females total; LG5-14 XY, *n* = 10 families, 115 males and 83 females total; *χ* ^2^ test for divergence from expected 1:1 sex ratio: *, *p* = 0.023, *χ* ^2^ = 5.2; ***, *p* < 0.0001, *χ* ^2^ = 15.2; LG13 ZW and LG5-14 XY family sex ratio differ from each other, *p* = 0.0053, Welch’s unequal variances *t-*test). **b** Penetrance by sex determination system, not significant

Previous studies have demonstrated that polygenic sex determination systems in cichlids produce skewed sex ratios. In a survey of species from Lake Malawi, families with sex determined by an XY system had equal sex ratios, while those with a ZW system generally had a female-biased sex ratio [22]. In families where both XY and ZW systems segregated, a 1:3 (male:female) sex ratio is expected [22]. In some Lake Victoria cichlids, sex determination factors on supernumerary chromosomes also produce female-skewed sex ratios [32]. Comparing the sex ratios of our LG5-14 XY families and LG13 ZW *A. burtoni* families reveals an intriguing and significant pattern: XY families have a male-biased sex ratio, and ZW families have a female-biased sex ratio (Fig. 3a). Because we only identified a few putative LG13 XY families, we do not test for significant divergence in sex ratio or penetrance.

Comparing penetrance of each sex determination allele, we find no significant differences (Fig. 3b), and a mean penetrance of 88 % for all sex determination alleles. Incomplete penetrance could be explained by a number of factors, including incorrect phenotype data, recombination between our sex-linked markers and the sex determination polymorphism, or sex reversal. If sex reversal is the case, it presents an alternative hypothesis regarding the interpretation that both W and Y alleles are present at LG13. The presence of a W allele has greater support based on replication with a number of dams, but only two sires were found to have their LG13 alleles segregate with sex of their offspring. Phenotypic males in some families with LG13 ZW dams occasionally carry a W haplotype at tested markers. For example, see Family 3, where two males exhibit the W-associated haplotype at LG13 (Additional file 6). Uncommon males that are genetically female (ZW at LG13) would have their LG13 alleles co-segregate with the sex of their offspring, lending the appearance of an XY system even though a distinct, male-determining Y allele might not exist at the locus.

### Genetic intervals for sex loci

Incomplete penetrance makes it difficult to determine mapping intervals via recombination breakpoint mapping, because it is unclear if genotype-phenotype mismatch at a particular marker is due to recombination or sex reversal. Though our ddRADseq families included relatively few individuals, we estimate genetic intervals based on strongly associated genome scaffolds. For the LG13 ZW locus, scaffolds spanning approximately 4.6 Mb show the highest association with sex (Fig. 1, Additional file 3). At LG5-14, a broad plateau of association spans 43 Mb of the linkage group based on data from the initial discovery family (Fig. 2). We further interrogated the association data with a sliding window analysis on LG5-14, to look for regions with the highest density of associated SNPs, which may correspond to the region of highest sequence divergence. Sliding window analysis reveals a broad peak of the highest density of associated SNPs on several scaffolds that align to LG5 of tilapia. In one family with LG5-14 XY sex determination, recombination breakpoints in one individual support that the region under this peak contains the sex determination locus (Individual 8, Family 18, Additional file 6; spanning scaffold 156 to scaffold 66).

### Candidate genes

Though precise genetic intervals are not clear, we examined gene content on the *A. burtoni* scaffolds with the highest association in our RAD family data (Additional file 8). Notably, genes with established roles in sex determination in multiple vertebrate species (e.g.*, sox3, dmrt, amh*, and gsdf) are not found on these scaffolds, suggesting the use of novel sex determination genes by *A. burtoni*. Here we highlight intriguing candidate genes on associated scaffolds based on known roles in sexual development or fertility.

#### LG13

The total length of sex-associated scaffolds at LG13 ZW is relatively small compared to those at LG5-14. One strong candidate for sex determination in the region is *cytochrome P450 17alpha-hydroxylase* (*cyp17a1*, scaffold 16), a key steroidogenic enzyme in the production of sex hormones in vertebrates [46]. Mutations of *CYP17A1* in humans produce low androgen levels and ambiguous or female external genitalia in XY individuals [47]. In amphibians (*Rana* frogs), *CYP17* has been repeatedly implicated as one of the key players in gonadal differentiation [48]. In the cichlid Nile tilapia, strong sexually dimorphic expression of *cyp17a1* is found during sexual differentiation [49]. Moreover, strong changes in *cyp17a1* expression accompany both natural sex reversal in field rice eel, a sequential hermaphrodite [50], and induced sex reversal in zebrafish [51]. Across species, activity of *cyp17a1* is associated with male sexual development, suggesting that a deleterious mutation to the gene in *A. burtoni* could produce a W allele inducing feminization of the gonad.

Another compelling candidate on LG13 is *type I bone morphogenetic protein receptor* (*bmpr1a*, scaffold 467), required for *Amh-*induced regression of female gonadal tissues in mammals; targeted disruption of *Bmpr1a* in Mullerian ducts in mice leads to pseudohermaphrodite males retaining oviducts and uteri [52]. *Amh* pathway members have been identified as sex determination genes in multiple fish species [7, 13, 34], demonstrating the importance of the pathway in sex determination. An evolved *bmpr1a* loss-of-function mutation in *A. burtoni* could reasonably shift gonadal development towards ovarian development.

A few genes found in the sex-associated region of LG13 are involved in production or response to gonadotropins. *Luteinizing hormone beta* (*lhb*, scaffold 186) is a subunit of luteinizing hormone, a gonadotropin secreted from the pituitary to direct gonadal development in fishes [53]. Notably, knock-out of *lhb* in zebrafish (*Danio*) produces female infertility via oocyte maturation defects, while testes and male fertility are normal, providing a sex-specific role for the gene in reproductive biology in fish [54]. *Ventral anterior homeobox 1* (*vax1*, scaffold 16) is essential for expression of gonadotropin-releasing hormone, with loss of *Vax1* in mice leading to hypogonadism and infertility in both sexes [55]. *Glial cell line derived neurotrophic factor family receptor alpha 1* (*gfra1*, scaffold 186) is expressed in spermatogonial stem cells in fishes in response to gonadotropins [56].

#### LG5-14

The sex-associated region at LG5-14 XY is broad, and thus we focus consideration of candidate genes on scaffolds on the region of LG5-14 with the highest density of sex-associated SNPs (Fig. 2, Additional file 8). Perhaps the most interesting candidate in the region is *wingless-type MMTV integration site family member 4* (*wnt4*, scaffold 156), required for female development in vertebrates, and considered one of the central female-promoting factors in sex differentiation networks [57–59]. While loss of *Wnt4* in mice causes partial female-to-male sex reversal [57], and there has been at least one case of sex reversal in an XY human carrying a *WNT4* duplication [60], to date *wnt4* has not been implicated as the primary sex determination gene in any species. *wnt7a* (scaffold 526) is also found in the XY region on LG5-14 in *A. burtoni*, and has been implicated in sexually dimorphic gonad development in mammals [61].

*R-spondin 4* (*rspo4*, scaffold 351) is a member of the R-spondin gene family, where *rspo1* is also a key member of vertebrate sex determination networks, with mutation in humans producing XX sex reversal [59, 62]. While a direct role for *rspo4* in sex differentiation has not yet been demonstrated, it exhibits sexual dimorphic expression in mammalian gonad, with higher levels in the ovary [63].

Multiple genes in the LG5 XY region modulate fundamental aspects of gonadal development. *Bone morphogenetic protein 7* (*Bmp7*, scaffold 9) regulates germ cell proliferation in mouse gonad [64], and has been implicated in gonadal divergence in chickens [65]. Further, *bmp7* is involved in androgen-dependent development of secondary sexual characteristics in medaka [66]. *GATA binding protein 2* (*gata2*, scaffold 9) is a zinc finger transcription factor that is expressed in a sexually dimorphic fashion during mouse gonadogenesis [67], and is known to regulate the expression of gonadotropins including *lhb* [68, 69], found in the LG13 ZW interval. *Cyp27b1* (scaffold 66) encodes *25-Hydroxyvitamin D3 1-alpha-hydroxylase*, the rate-limiting enzyme in synthesis of the active form of Vitamin D3; female *Cyp27b1* knock out mice have extensive reproductive defects, including delayed puberty, uterine hypoplasia, altered estrogen and gonadotropin levels, and infertility [70–72].

### A second chromosome fusion

Because our ddRADseq haplotype data was able to resolve the linkage groups involved in one of two the *A. burtoni* chromosome fusions relative to other cichlids [44], we attempted to identify the other known fusion by exploring linkage between markers in our RAD genotype data. Building an outcross linkage map with only 24 offspring from RAD family 1 produced a poorly resolved linkage map, with 30 linkage groups containing greater than five markers. Though the map was very rough, it identified candidates for linkage group fusions that were then examined by manual comparison of haplotypes. Markers aligning to two tilapia linkage groups, LG8-24 and LG16-21, were linked in our map output. (The nomenclature for both LG8-24 and LG16-21 in tilapia does not indicate fused chromosomes, but is instead a historical artifact: the standard *O. niloticus* linkage map originally had 24 linkage groups [73], but later improvement of the genetic map identified 22 linkage groups [42], collapsing the former LG8 and LG24 into a single linkage group, as well as LG16 and LG21.) We find continuous haplotypes spanning LG8-24 and LG16-21, with significant linkage between markers aligning to the two linkage groups (LOD = 10.3, Additional file 4), supporting the fusion of the two associated chromosomes in *A. burtoni* relative to other cichlids. Maintaining the community naming convention based on the original *O. niloticus* linkage map, this linkage group would be given the rather cumbersome designation LG8-24-16-21. A previous report suggested that one of the fusions in *A. burtoni* consisted of LG15 and LG19 relative to other cichlids [31], but we find no evidence of linkage between markers mapping to those linkage groups (LOD = 0.8, Additional file 4). This discrepancy may stem from interchromosomal rearrangements more complex than simple fusions, where different portions of a particular tilapia LG map to different *A. burtoni* LGs. Thus, use of different genetic markers could provide patterns suggesting fusion of different LGs.

### Comparisons among cichlid sex determination systems

A previous study inferred the mode of sex determination system in *A. burtoni* using hormonal sex reversal [40], in a line derived from the same lab strain we use here. Estradiol sex reversal of males and subsequent testcrosses strongly indicated an XY sex determination system, and ruled out a ZW system [40]. These results are consistent with our mapping data, because it is quite possible that only the XY sex determination system was present in the four families used for sex reversal experiments. Indeed, bottlenecks during transfer of the *A. burtoni* lab strain between labs may inadvertently lead to complete loss of some sex determination alleles. Concurrent mapping experiments by another group using the same lab line as the sex reversal experiments above identified co-segregation of sex with markers on LG5 indicating an XY system [74], completely consistent with the mapping results we describe here. While the concurrent study found no association of sex with LG13, it did identify an association of sex with LG18 in the offspring of a pair of wild-caught individuals from a Lake Tanganyika estuary [74]. Together, our differing but overlapping results support the presence of complex, polygenic, and potentially rapidly evolving sex determination in *A. burtoni*.

A number of sex determination loci have been identified in cichlids. In tilapia, sex determination has been associated with LG3, LG20, LG23, and two distinct loci on LG1, depending on the species and population [30, 35, 36, 75]. In Lake Malawi haplochromine cichlid species, LG3, LG5, LG20, and two loci on LG7 have been associated with sex determination [21, 23]. In haplochromine cichlids from Lake Victoria, LG2, LG5, and supernumerary B chromosomes have been associated with sex [31, 32]. Here we identify two loci impacting sex determination in the riverine haplochromine *A. burtoni,* LG5-14 and LG13. While this is the first time LG13 and LG14 have been associated with sex determination, it is intriguing that LG5 has now been associated with haplochromine sex determination in *A. burtoni*, Malawi, and Victoria cichlids. To provide finer scale comparison of sex association with LG5, we identified *A. burtoni* scaffolds containing sex-associated markers from other studies (Fig. 2b), revealing overlap of sex loci among multiple species. As on-going mapping studies identify specific polymorphism underlying sex determination in each context, we should be able to distinguish reuse of ancestral polymorphism versus repeated *de novo* evolution of sex determination on LG5. However, both W [21, 22] and Y (this study; also suggestive patterns in two families in [22]) acting alleles have been associated with LG5, suggesting at least two evolutionary events. LG5 has been dubbed a “speciation chromosome” because of the number of adaptive and species-delineating phenotypes mapped there, including pigmentation, vision, jaw shape, and tooth shape [76], and it may exhibit dynamic evolution of sex determination with both W and Y alleles now clearly identified. The identification of the LG5-14 fusion in *A. burtoni* should provide additional context to consider the role of this chromosome in adaptation and speciation, particularly in comparing the evolutionary trajectory of LG14 among lineages with and without the fusion. Indeed, the LG5-14 fusion in *A. burtoni* may represent karyotype evolution driven in part by the presence of a sex determination locus on LG5. For example, if novel alleles under strong sexually antagonistic selection arose on LG14, the resulting sexual conflict could have been resolved by fusion to the LG5 sex chromosomes, ensuring that sex-specific fitness benefits were only expressed in the appropriate sex.

### Evolutionary context

Evolutionarily, the genus *Astatotilapia* represents an immediate outgroup to the adaptive radiations of cichlid species in Lake Malawi and Lake Victoria [41, 77], providing important context for studies of sex determination evolution in those flocks. Ongoing studies may reveal if the sex determination locus on LG5/LG5-14 is the first found both within and without the lake adaptive radiations, and/or shared among radiations. Additionally, a recent report implicated the same region of LG5 as one of a handful of genomic islands of speciation in *Astatotilapia* species, based on sequence divergence between ecomorphs ([78]; Fig. 2b). One hypothesis is that the signal of divergence at LG5 in that study is due to divergence related to the sex determination locus, with the LG5 XY system potentially present in one ecomorph and absent in the other.

Ultimately, our mapping results must be confirmed in natural populations. The recent discovery that the natural sex determination chromosome in zebrafish (*Danio*) was lost in lab strains urges caution in interpreting genetic mapping of sex determination in domesticated fish [28]. More recently, large-scale changes in gene expression were identified after only a single generation of domestication in trout [79], suggesting that domestication can act rapidly on the phenotypic output of the fish genome. The standard lab strain of *A. burtoni* we use here has been maintained in the lab for nearly four decades [80], representing over 100 generations in captivity in which loss or gain of sex determination alleles could have taken place. Indeed, recently collected stocks of *A. burtoni* exhibit a number of phenotypic differences from the classic lab strain [81], suggesting underlying genetic differences between wild and lab strain *A. burtoni*.

In general, the evolutionary pressures that maintain polygenic sex determination systems remain unknown. We favor the hypothesis that the multiple sex genotypes produced by polygenic sex determination may provide alternative fitness strategies, for example, alternate sex genotypes may be associated with differences in color (yellow vs. blue male morphs) or behavior (potential sneaker vs. territorial mating strategies) in *A. burtoni* [82, 83]. In any case, *A. burtoni* should serve as an excellent model for understanding the outcomes of polygenic sex determination for primary and secondary sexual characteristics. The availability of genome editing tools in the species permits mechanistic dissection of genetic variation [84, 85], while decades of research in the species provides a wealth of information regarding sexually dimorphic behavior, physiology, and related gene expression [38, 81, 86–89].

## Conclusions

Examples of polygenic sex determination are accumulating across taxa [3], and particularly among cichlid fish species. Here we present evidence for polygenic sex determination in the cichlid *A. burtoni*, a longstanding model of sex-related physiology and behavior, and a close relative of the explosive adaptive radiations of species in Lake Malawi and Lake Victoria. Our results strongly indicate an XY system on LG5-14 and a ZW system on LG13. Additionally, inheritance in some families indicates a Y allele on LG13, which would support the presence of an XYW system; however, occasional apparently sex reversed individuals make the conclusion that both W and Y alleles are present at LG13 tentative. The locus on LG13 represents a novel association with sex, while LG5 has been implicated in sex determination, adaptation, and speciation in a number of contexts. Our results add to the evidence that LG5 may have played an unusual role in cichlid diversification, including fusion of LG5 and LG14 in *A. burtoni*. Ongoing investigation of sex determination in *A. burtoni* aims to identify the sex determination genes and understand their action and interaction on primary and secondary sexual characteristics. Understanding the complex genetics underlying sex determination in *A. burtoni* should provide additional insight and context to an already fundamental model of sexual dimorphism and behavioral evolution.

## Methods

### Husbandry and phenotyping

Fish husbandry and procedures were performed under Institutional Animal Care and Use Committee approved protocols at North Carolina State University (Roberts Lab) and Stanford University (Fernald Lab), with all fish originating from Fernald Lab lines. Breeding tanks were set up with a single male and multiple females. The species is a maternal mouth brooder, allowing parentage to be determined. Fertilized eggs were collected in the first week post-fertilization and tumbled in flasks until fry were free swimming. At approximately 6 weeks post-fertilization, families were transferred to 50 gal aquaria until sexual maturity, between 4 and 5 months of age. Fish were euthanized in a bath of 250 mg/L tricaine methanesulfonate (Tricaine-S, Western Chemical) and dissected for gonad collection. Gonads clearly containing eggs were scored as female, while other gonads were squashed between slides and visualized under a microscope to determine the presence of sperm (male) or immature ovarian follicles (female). Sex ratio was calculated as the number of male offspring divided by the total number of offspring in the family.

### Genotyping

DNA was extracted from finclips taken from parents and offspring using standard methods (GeneJET Genomic DNA Purification Kit, Thermofisher). ddRADseq libraries were built as previously described [90] using *SphI* and *MluCI* enzymes, and sequenced on a single Illumina HiSeq lane with 100 bp paired-end reads (North Carolina State University Genomic Sciences Laboratory). Reads were de-multiplexed, allowing zero mismatches with the variable length barcode sequence [90]. Barcodes were trimmed from each read, and 10-N bases were trimmed from the end of each read, where N is the number of bases in the barcode. Thus, all reads were exactly 90 bases long for alignment. Reads were aligned to reference (*A. burtoni* v1; [41]) using m = 1 and genotypes called in *Stacks* [91]. For further analysis we used markers with genotypes called in at least 20 of 24 offspring for RAD family 1, and 15 of 17 offspring for RAD family 2, using a minimum stack depth of 10 to call homozygous genotypes. Association of ddRADseq markers with phenotypic sex was performed in PLINK [92], using Fisher’s exact test to determine *p*-values of association. Genotyping of SSR markers used PCR with fluorophore-labeled primers (Additional file 9) and separation on an Applied Biosystems 3730 capillary sequencer as previously described [21]. Fisher’s exact test was used to determine significant co-segregation of parental alleles with offspring sex. Where a significant association was found in a family, penetrance was calculated as the proportion of offspring with the expected genotype-phenotype association at the marker with the lowest Fisher’s exact test *p* value. We note this is an imperfect measure of penetrance because linked markers are used rather than causative sex determination polymorphisms. *Onemap* was used to order RAD genotypes into linkage groups using outcross mapping, linking markers at LOD >4 and a maximum recombination fraction of 0.5 [93].

### Comparative genomics

The *A. burtoni* genome (v1; [41]) was partitioned into non-overlapping 5 kb sequence windows and mapped to reference genomes (Tilapia_v1.1 and Mzebra_v0; [41]) using the MEM algorithm implemented in bwa (v. 0.7.5a; [94]). The resulting bam file was filtered to include only sequences mapping to a single coordinate in the reference genome and with fewer than 5 suboptimal hits. Aligned sequences with less than 90 % sequence identity to the reference reference were also removed. This strategy allowed us to place 637 scaffolds from the unanchored *A. burtoni* genome onto the 22 assembled Tilapia linkage groups. An additional 99 *A. burtoni* scaffolds were confidently placed onto unmapped contigs in the Tilapia assembly. The resulting alignments and association data were visualized using custom R scripts. For sliding window analysis, windows of 10 SNPs with 1 SNP step size were used, and the resulting line smoothed for visualization purposes.

## Additional files

Additional file 1: Alignment of *A. burtoni* scaffolds to anchored *O. niloticus* (Nile tilapia) genome. (PDF 725 kb)
Additional file 2: Alignment of *A. burtoni* scaffolds to anchored *M. zebra* genome. (PDF 619 kb)
Additional file 3: Association results of sex with RAD markers in two families. (XLSX 653 kb)
Additional file 4: Linkage tests and RAD marker haplotypes spanning two chromosome fusions in two families. (XLSX 110 kb)
Additional file 5: Expanded summary table of association of genetic markers with sex in families, including genetic markers used. (XLSX 53 kb)
Additional file 6: SSR marker data at LG13 and LG5-14 for 25 families. (XLSX 145 kb)
Additional file 7: SSR marker data at LG13 and LG5-14 for Family 19, with inferred Y haplotype and offspring sex genotypes. (XLSX 51 kb)
Additional file 8: Gene annotations for scaffolds on LG13 and LG5-14 associated with sex. (XLSX 440 kb)
Additional file 9: Primer sequence and scaffold location for SSR markers used. (XLSX 35 kb)

## Abbreviations

ddRADseq: Double-digest restriction site associated DNA sequencing
LG: Linkage group
LOD: Logarithm of odds
Mb: Megabases
SNP: Single nucleotide polymorphism
SSR: Simple sequence repeat
UNK: Unknown linkage group; portion of genome assembly not included on a linkage group
