# Supplementary figures and images for: Polygenic sex determination in the cichlid fish Astatotilapia burtoni
Source: BMC Genomics. 2016 Oct 26;17:835. doi: 10.1186/s12864-016-3177-1 (PMC5080751; doi:10.1186/s12864-016-3177-1)

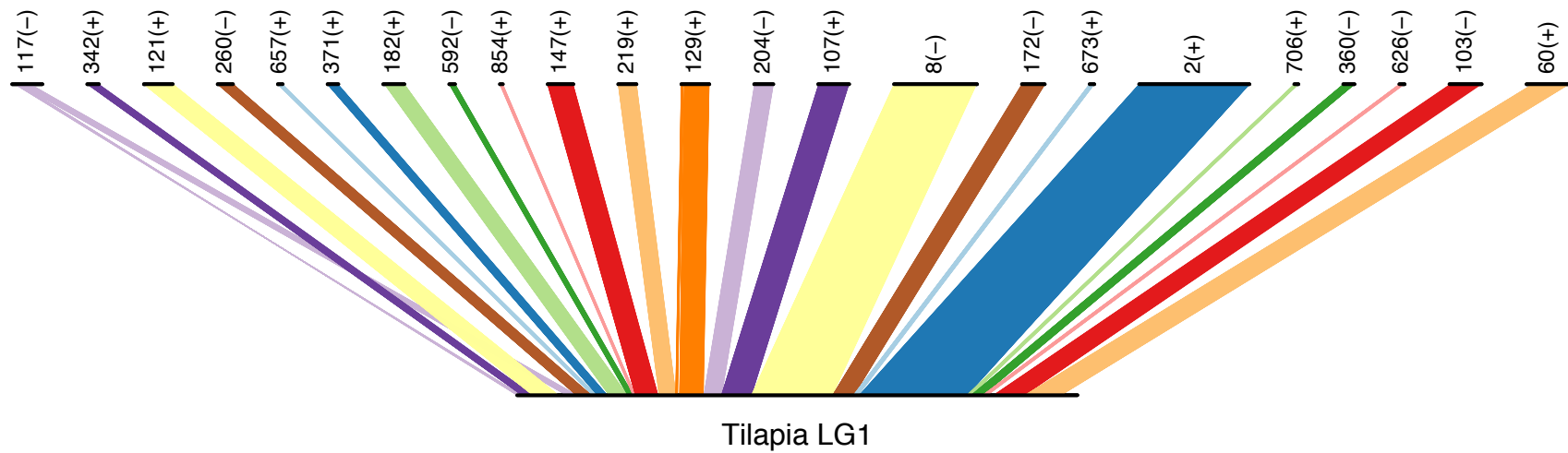

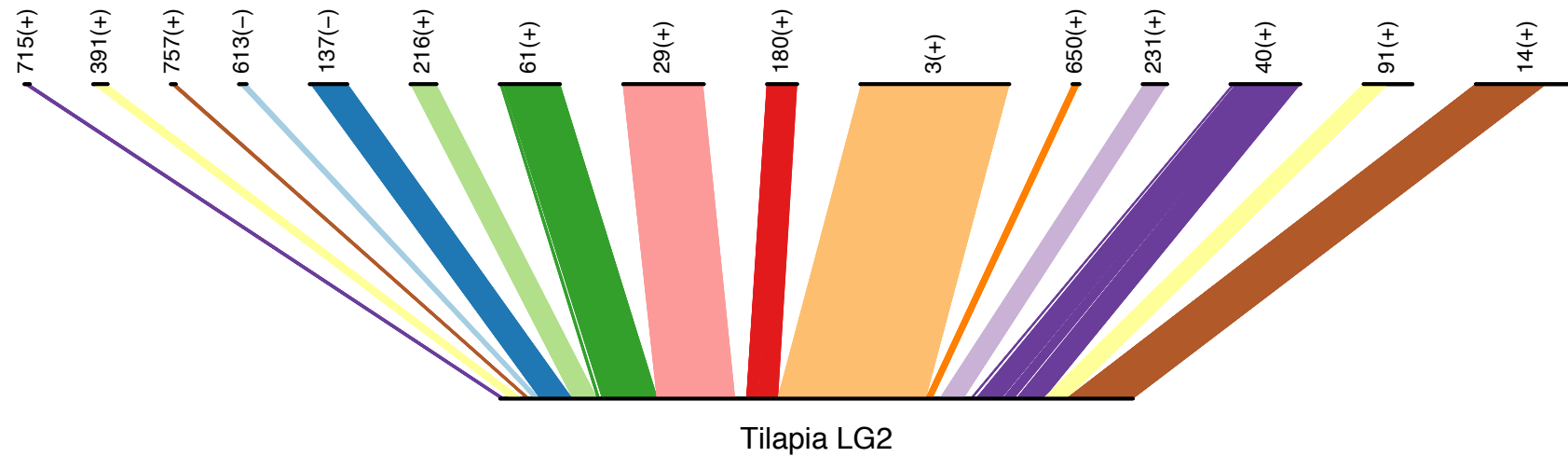

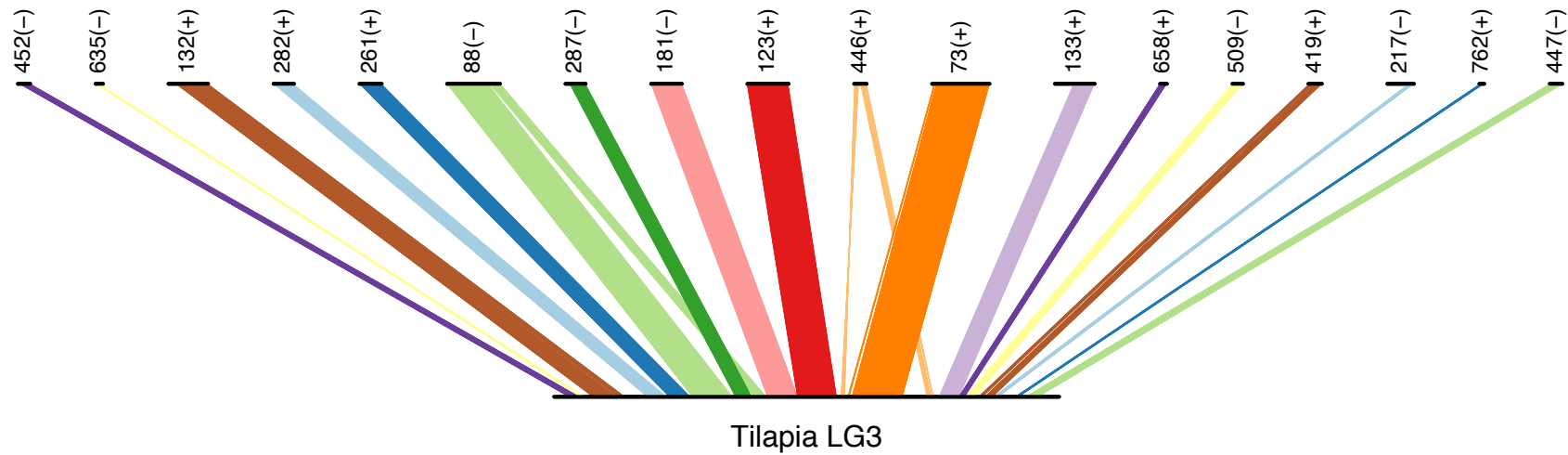

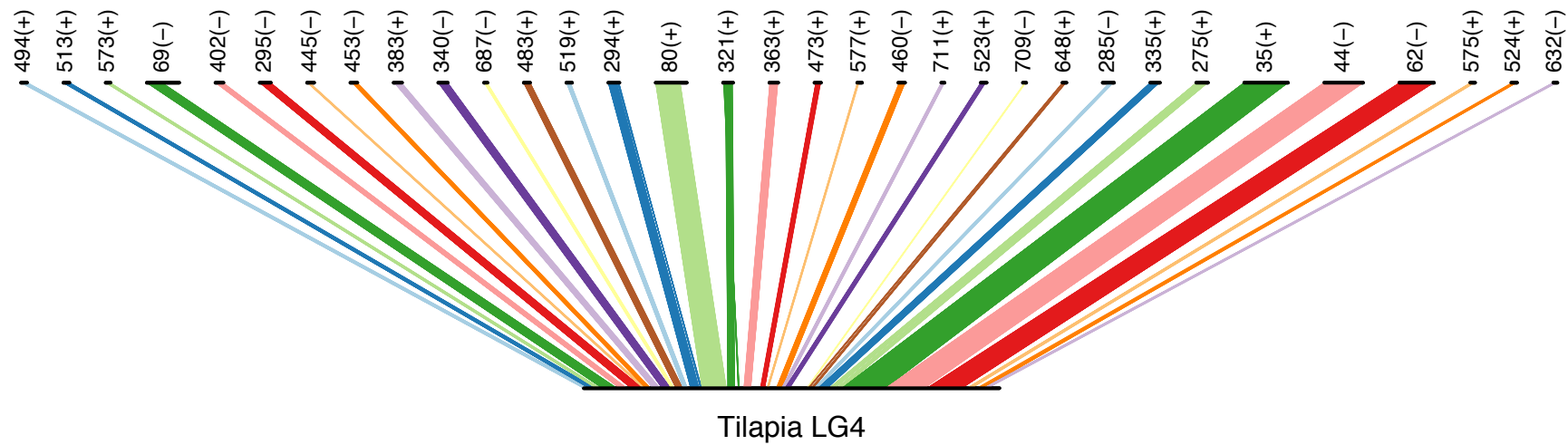

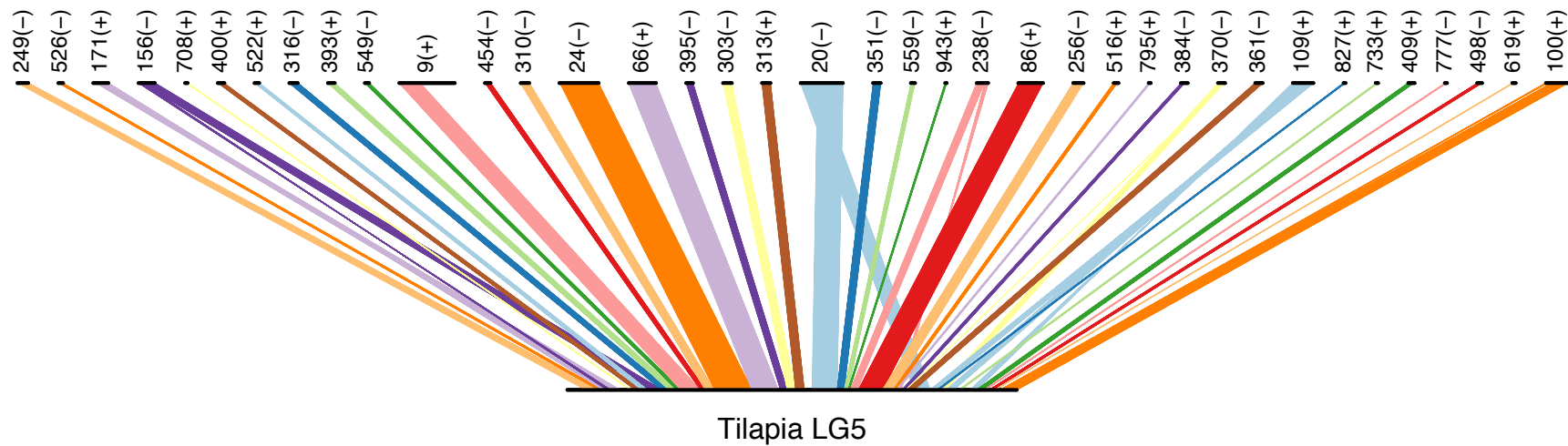

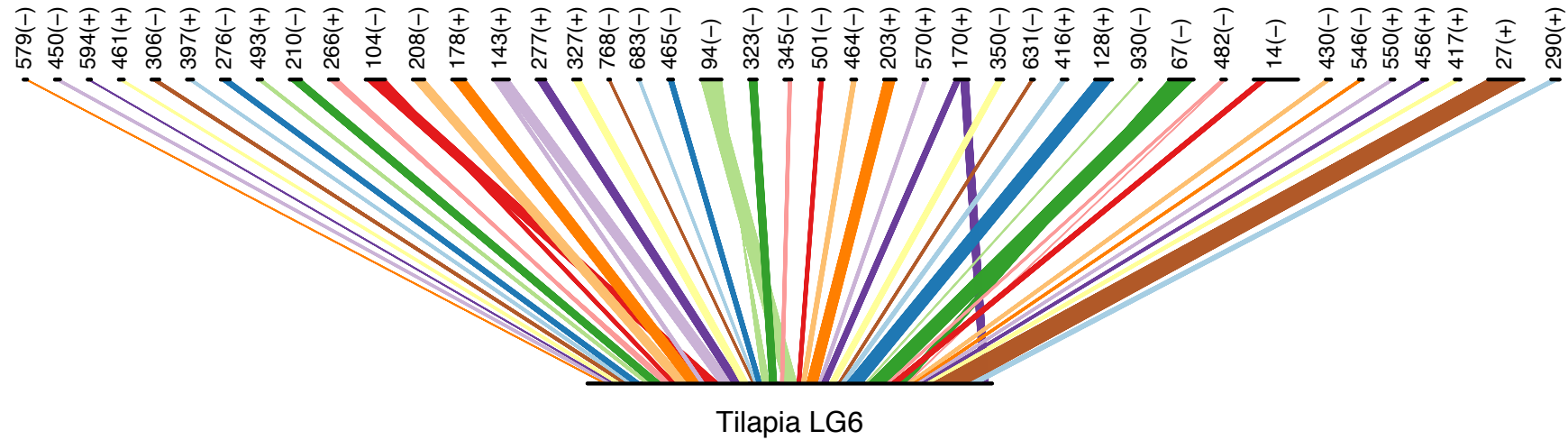

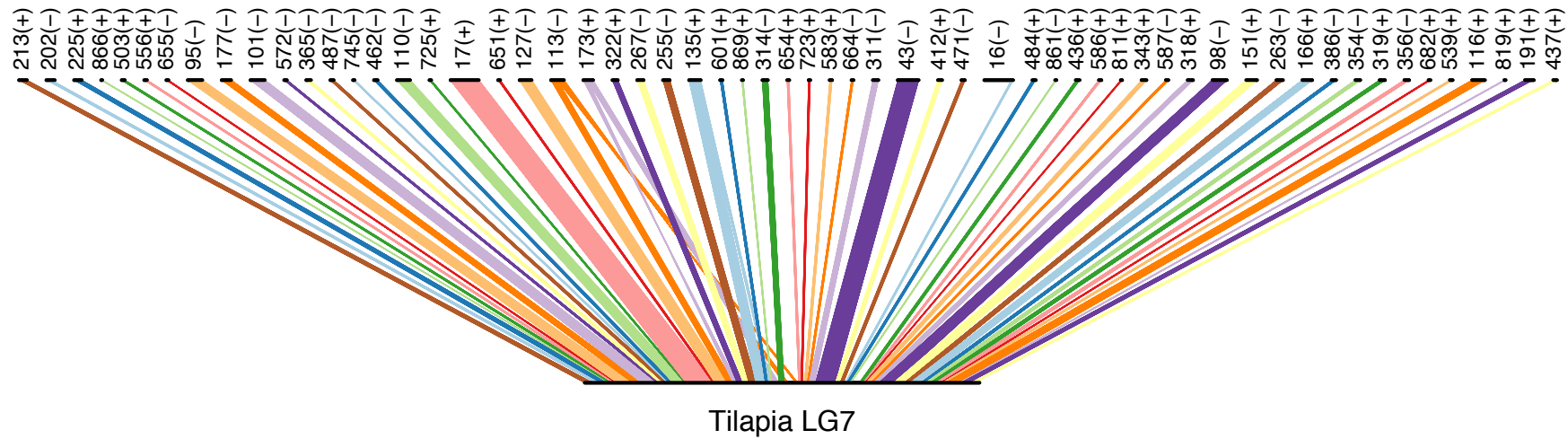

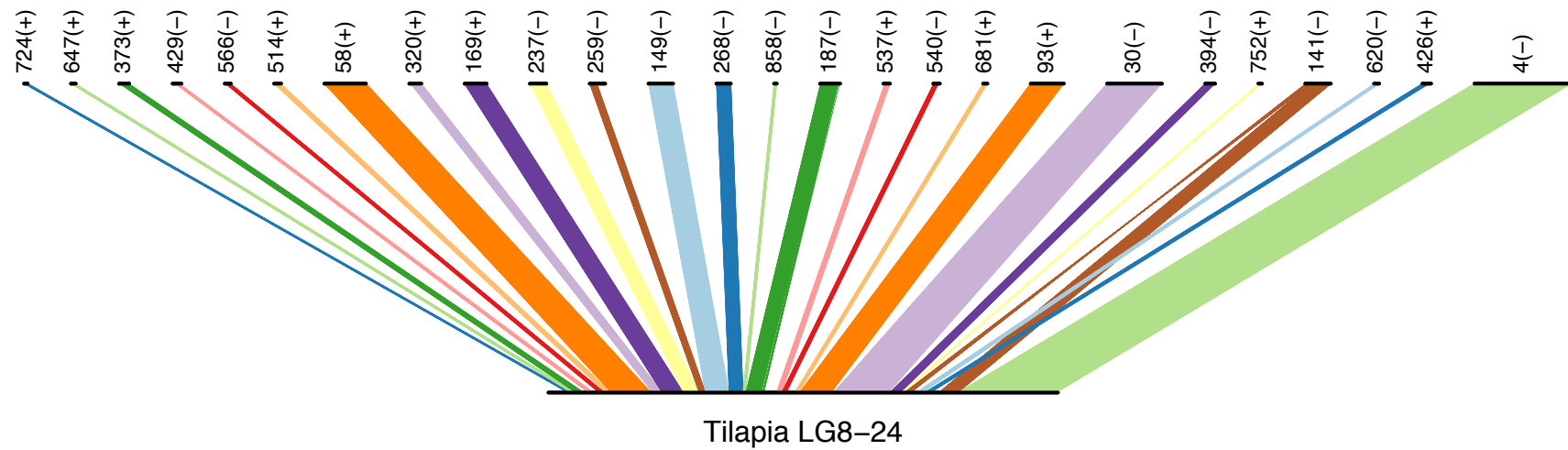

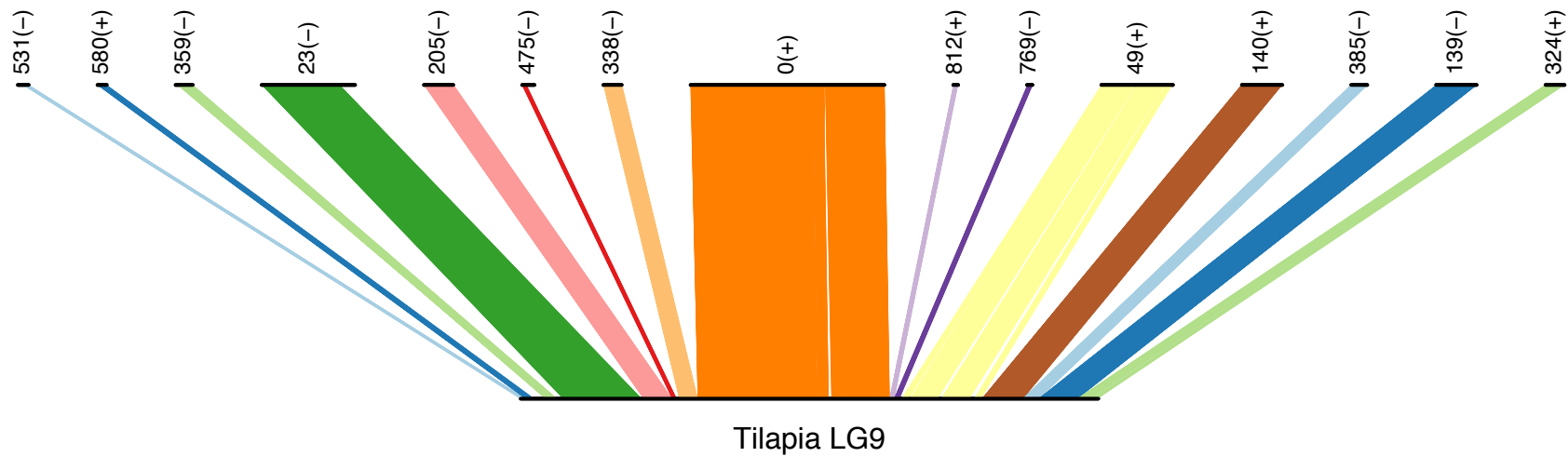

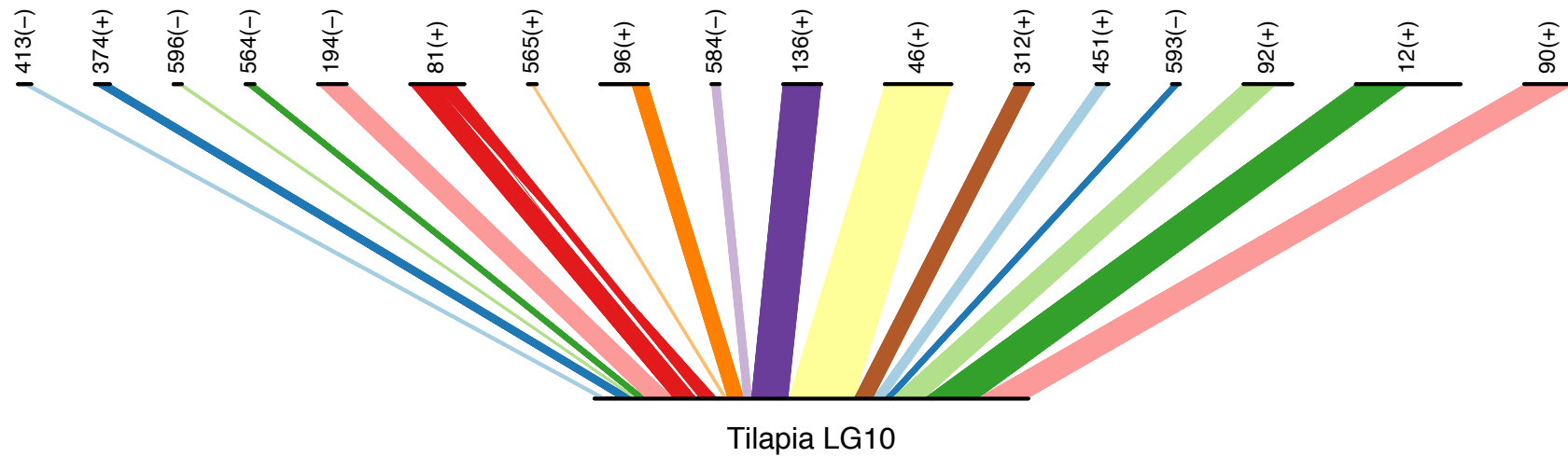

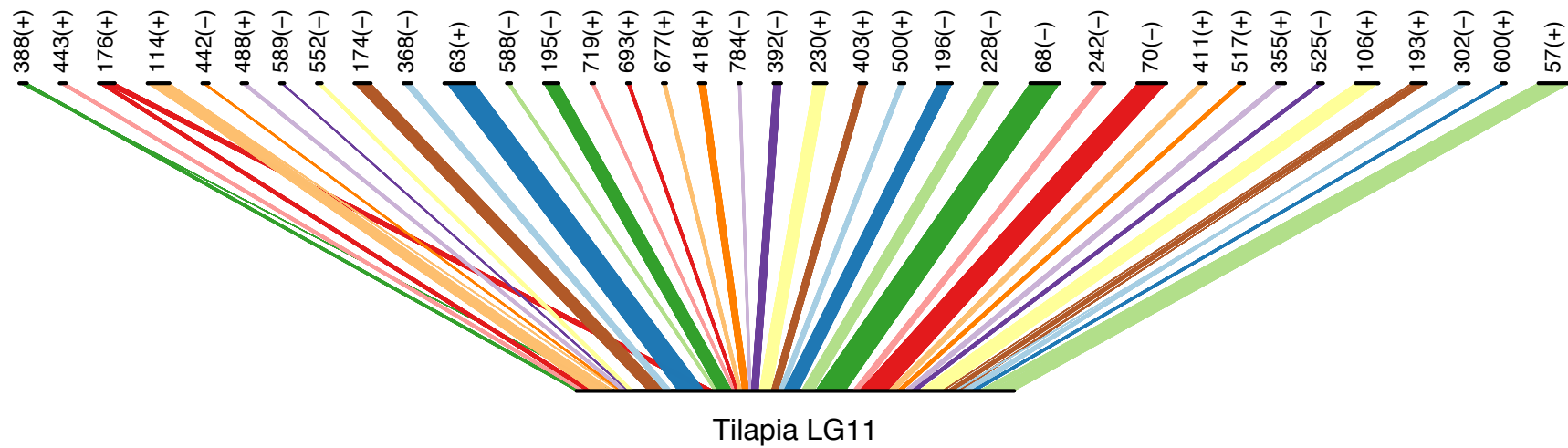

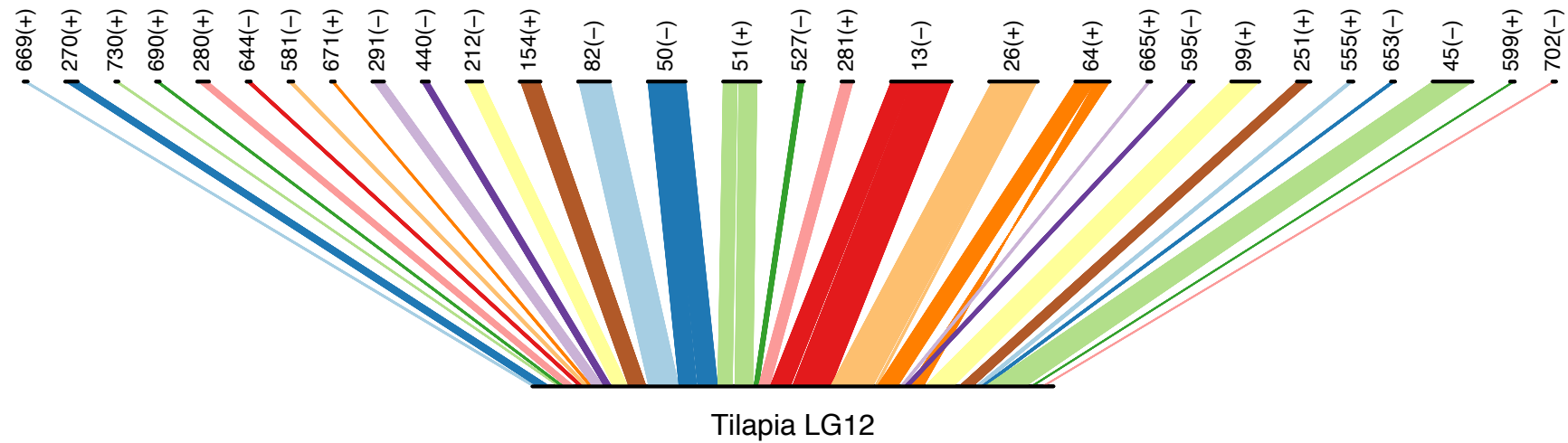

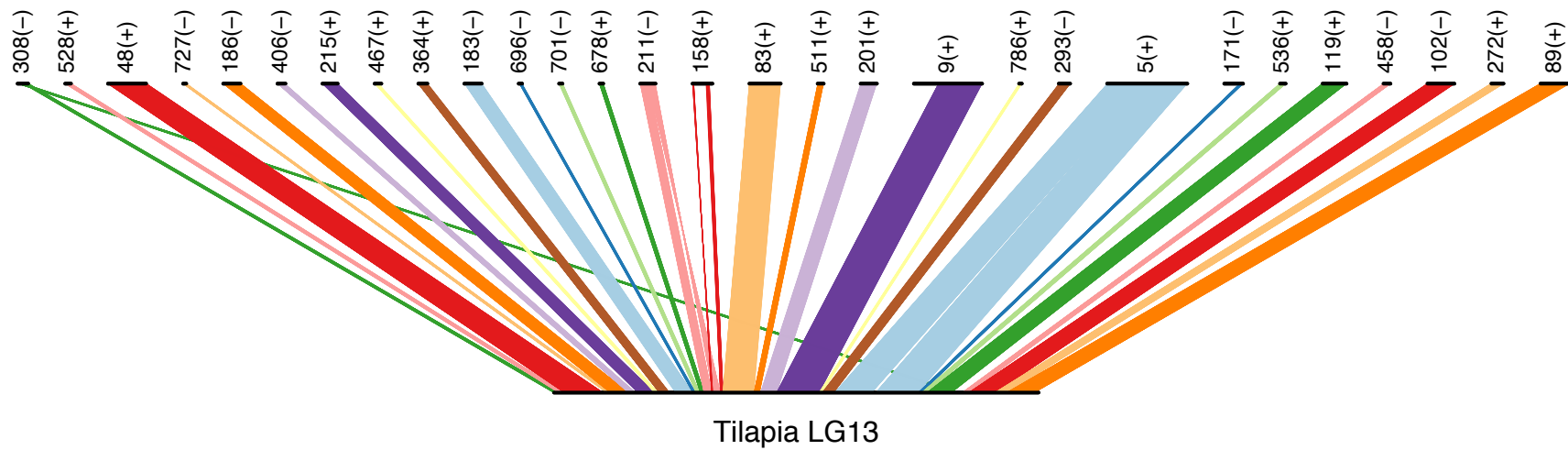

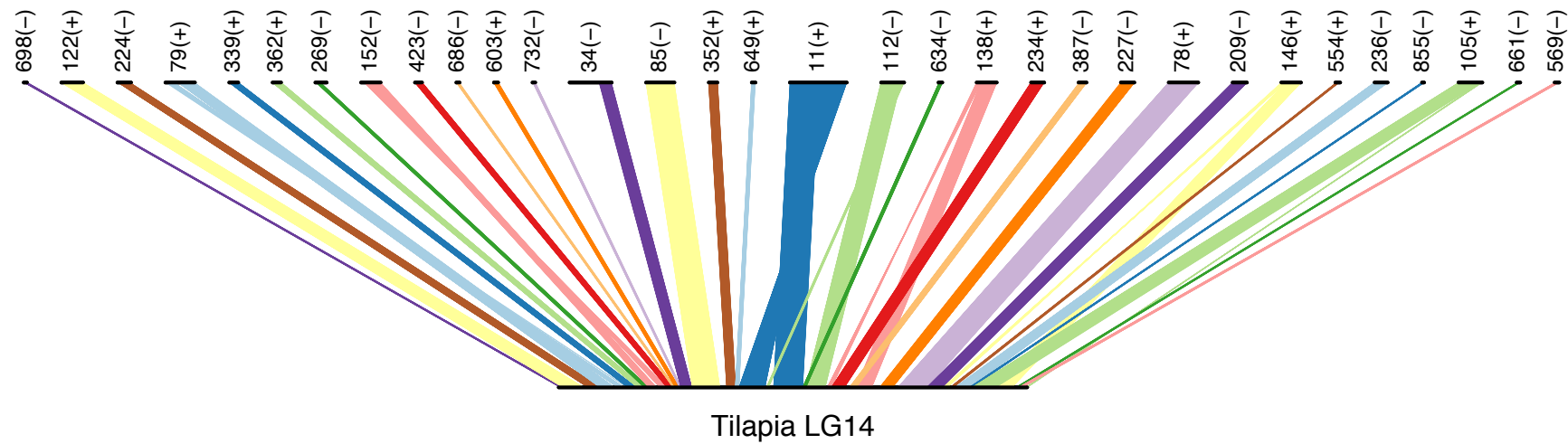

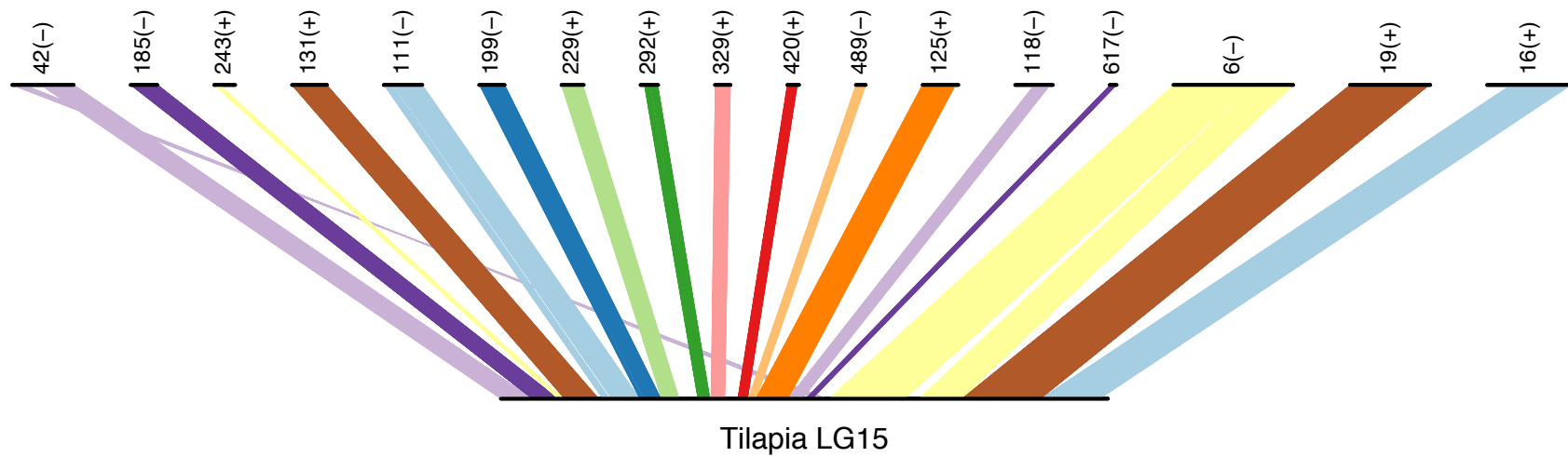

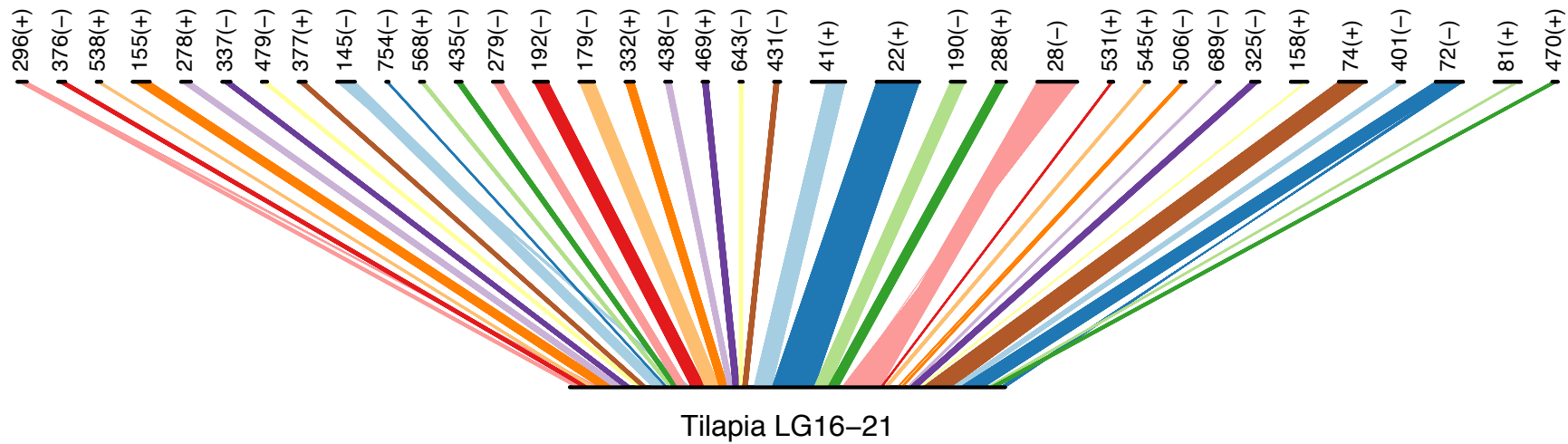

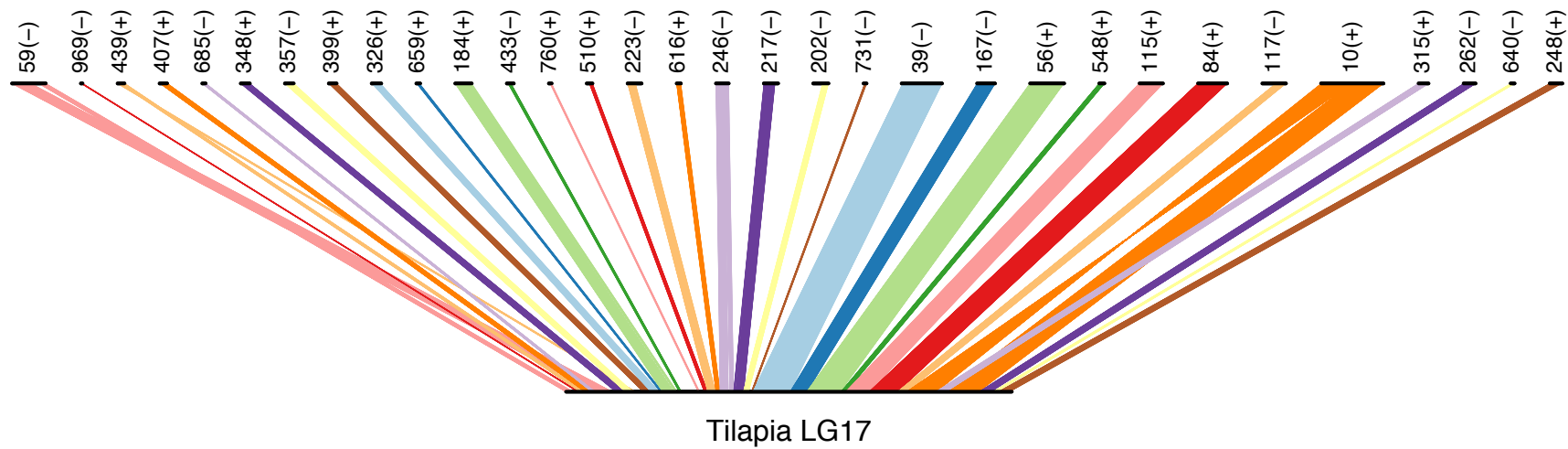

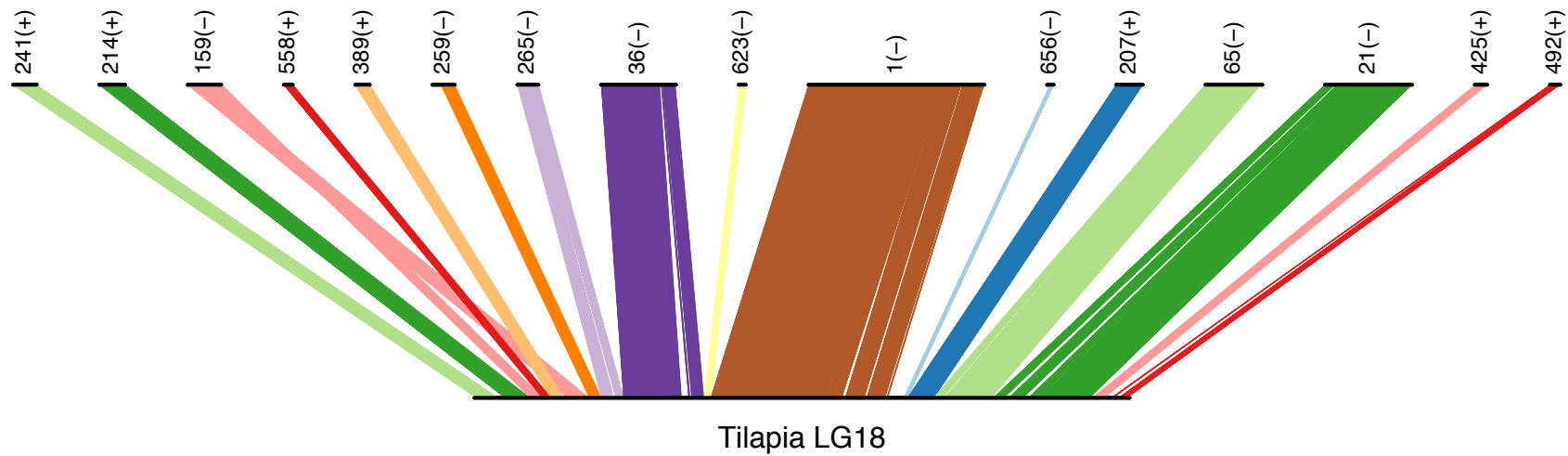

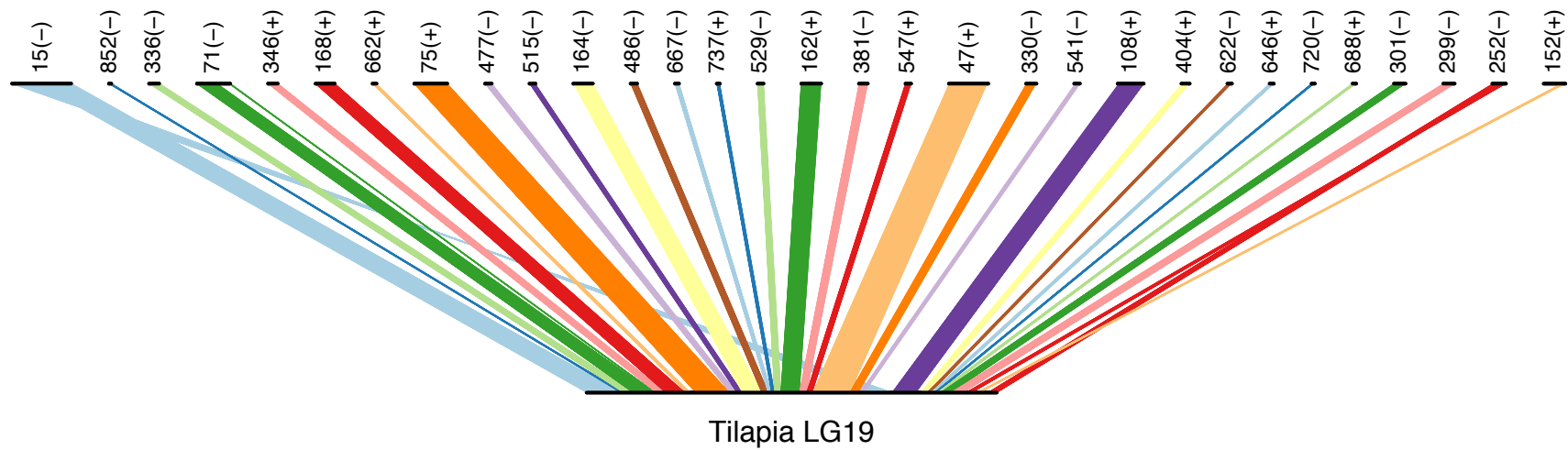

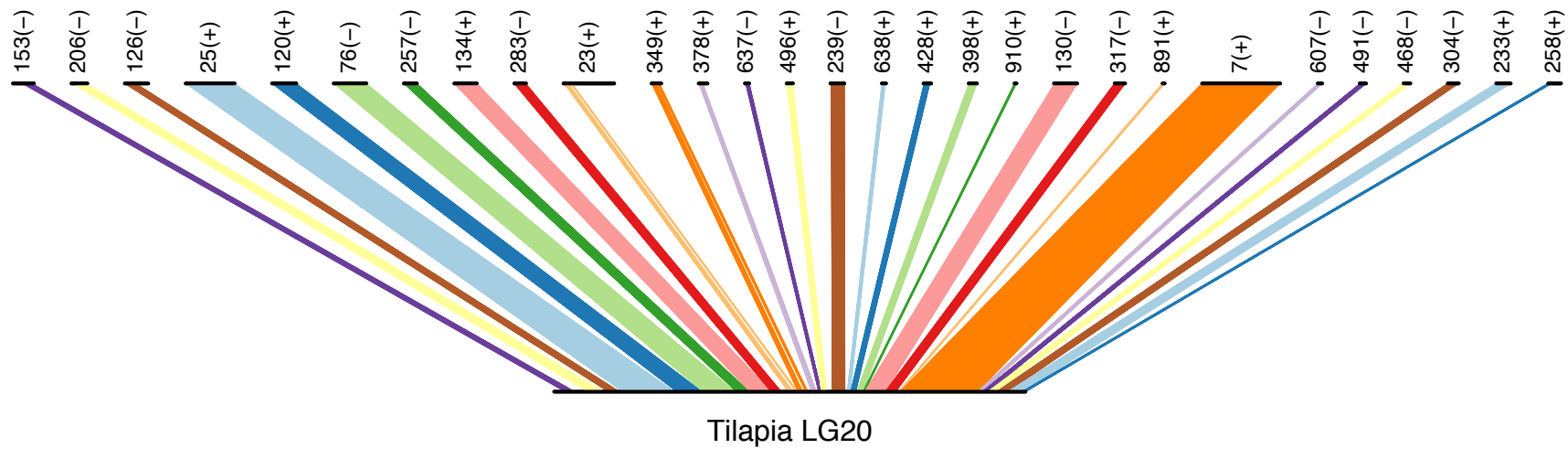

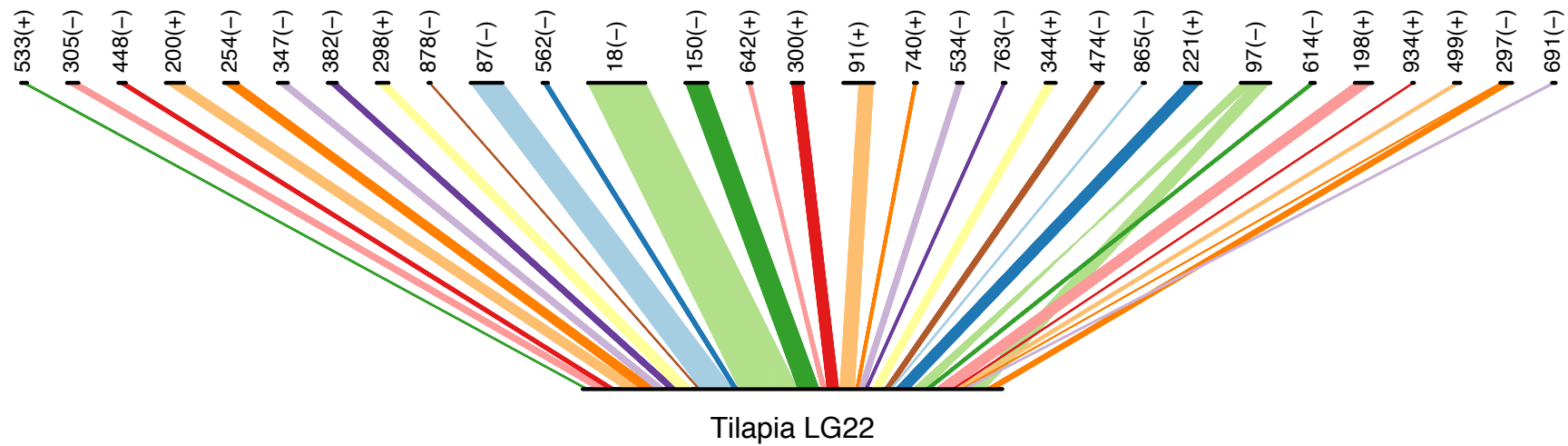

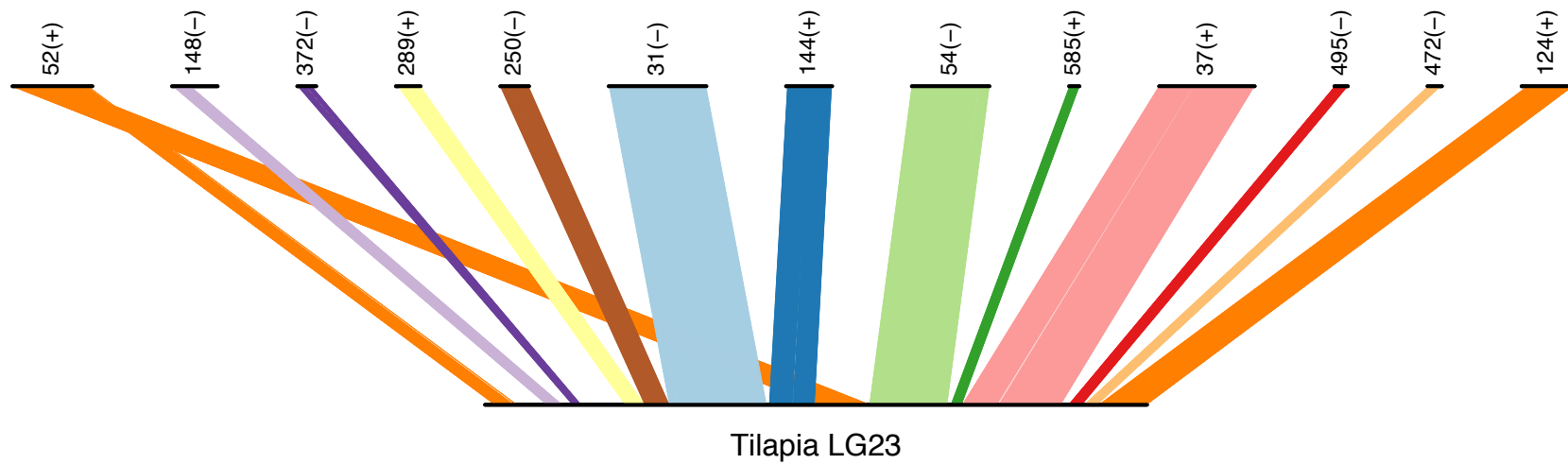

Supplement: Additional file 1: — Alignment of A. burtoni scaffolds to anchored O. niloticus (Nile tilapia) genome. (PDF 725 kb) [file 12864_2016_3177_MOESM1_ESM.pdf]
