# Supplementary material for: Polygenic sex determination in the cichlid fish Astatotilapia burtoni
Source: BMC Genomics. 2016 Oct 26;17:835. doi: 10.1186/s12864-016-3177-1 (PMC5080751; doi:10.1186/s12864-016-3177-1)
Supplement: Additional file 2: — Alignment of A. burtoni scaffolds to anchored M. zebra genome. (PDF 619 kb) [file 12864_2016_3177_MOESM2_ESM.pdf]

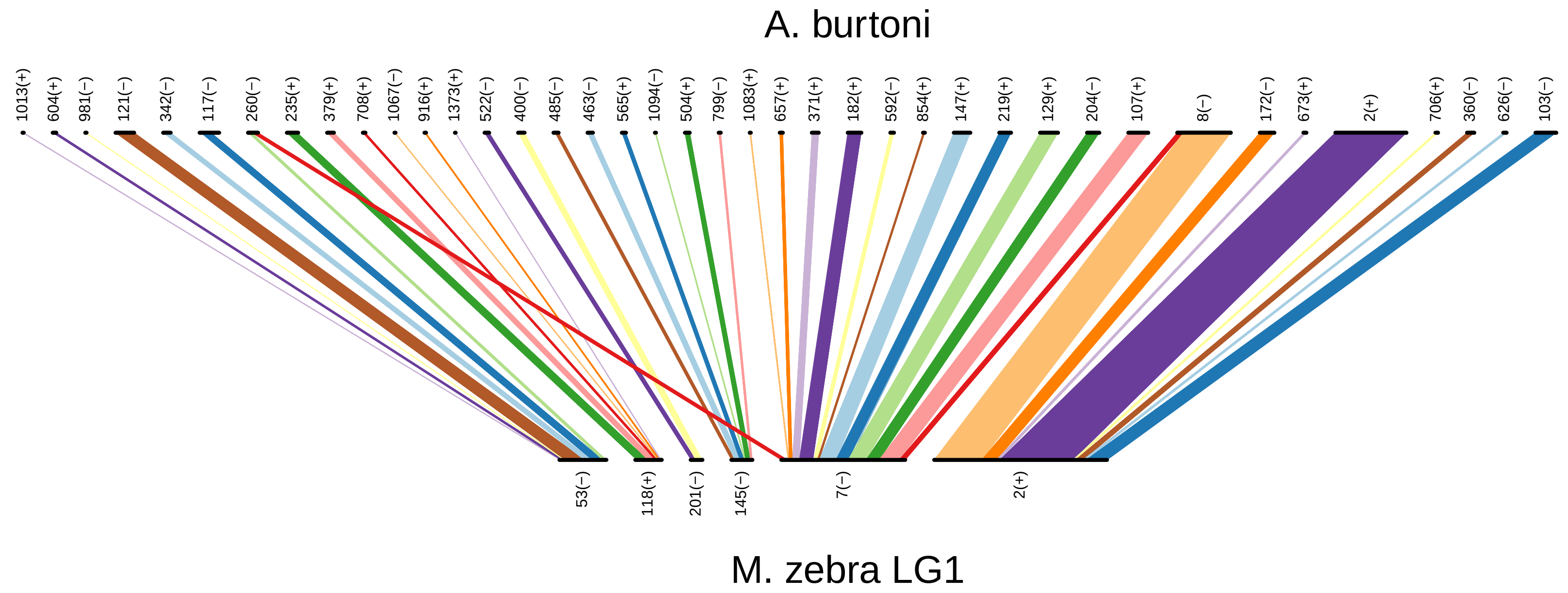
A. burtoni
1013(+)
604(+)
981(−)
121(−)
342(−)
117(−)
260(−)
235(+)
379(+)
708(+)
1067(−)
916(+)
1373(+)
522(−)
400(−)
485(−)
463(−)
565(+)
1094(−)
504(+)
799(−)
1083(+)
657(+)
371(+)
182(+)
592(−)
854(+)
147(+)
219(+)
129(+)
204(−)
107(+)
8(−)
172(−)
673(+)
2(+)
706(+)
360(−)
626(−)
103(−)
53(−)
118(+)
201(−)
145(−)
7(−)
2(+)
M. zebra LG1

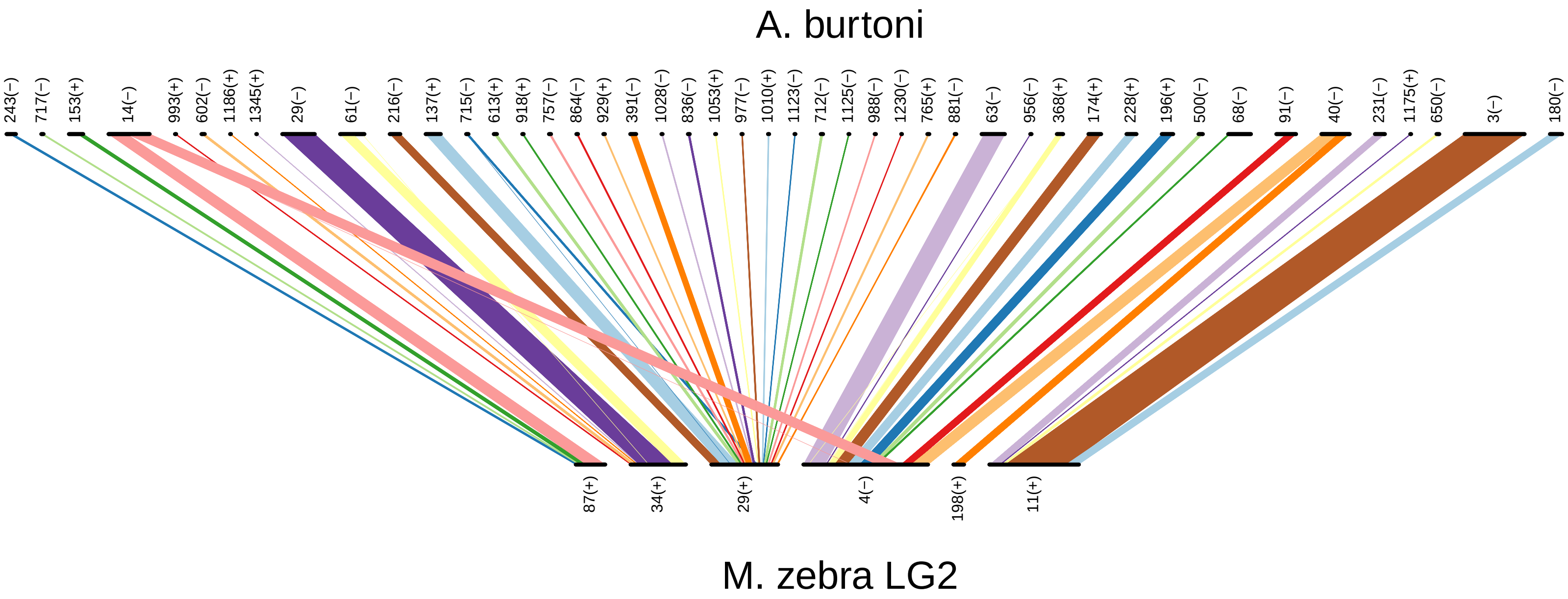
A. burtoni
243(−)
717(−)
153(+)
14(−)
993(+)
602(−)
1186(+)
1345(+)
29(−)
61(−)
216(−)
137(+)
715(−)
613(+)
918(+)
757(−)
864(−)
929(+)
391(−)
1028(−)
836(−)
1053(+)
977(−)
1010(+)
1123(−)
712(−)
1125(−)
988(−)
1230(−)
765(+)
881(−)
63(−)
956(−)
368(+)
174(+)
228(+)
196(+)
500(−)
68(−)
91(−)
40(−)
231(−)
1175(+)
650(−)
3(−)
180(−)
87(+)
34(+)
29(+)
4(−)
198(+)
11(+)
M. zebra LG2

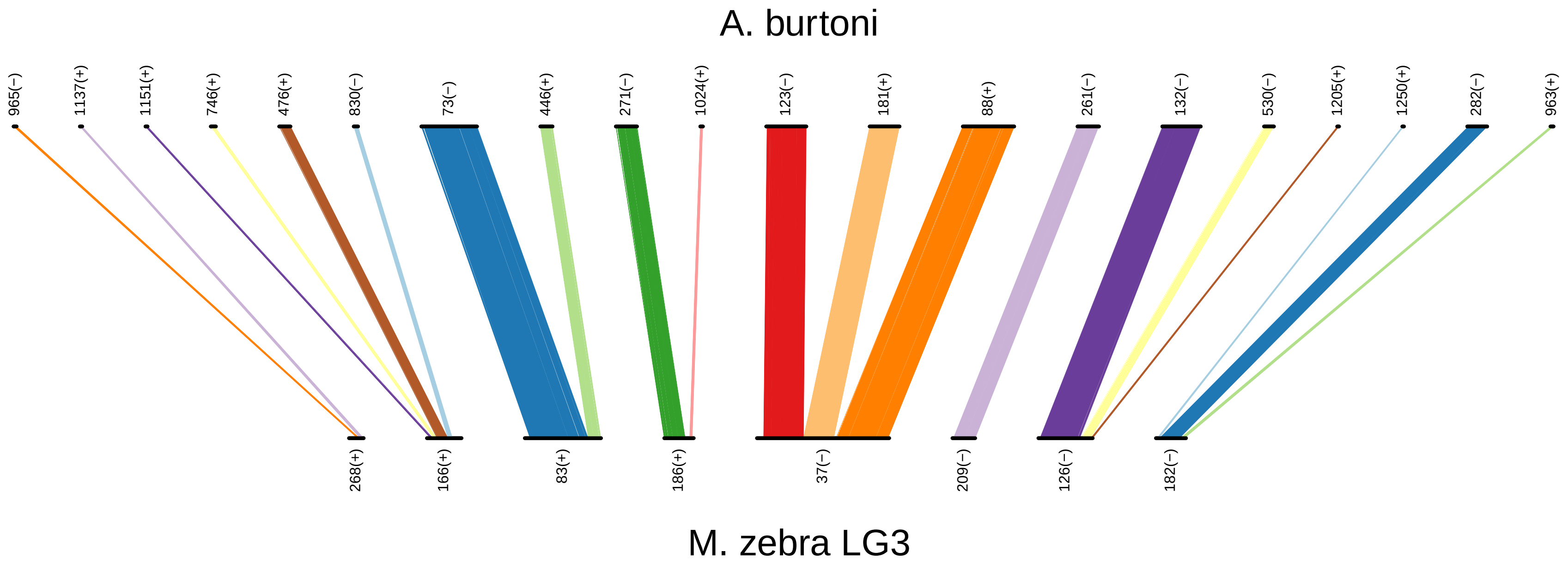
A. burtoni
965(−)
1137(+)
1151(+)
746(+)
476(+)
830(−)
73(−)
446(+)
271(−)
1024(+)
123(−)
181(+)
88(+)
261(−)
132(−)
530(−)
1205(+)
1250(+)
282(−)
963(+)
268(+)
166(+)
83(+)
186(+)
37(−)
209(−)
126(−)
182(−)
M. zebra LG3

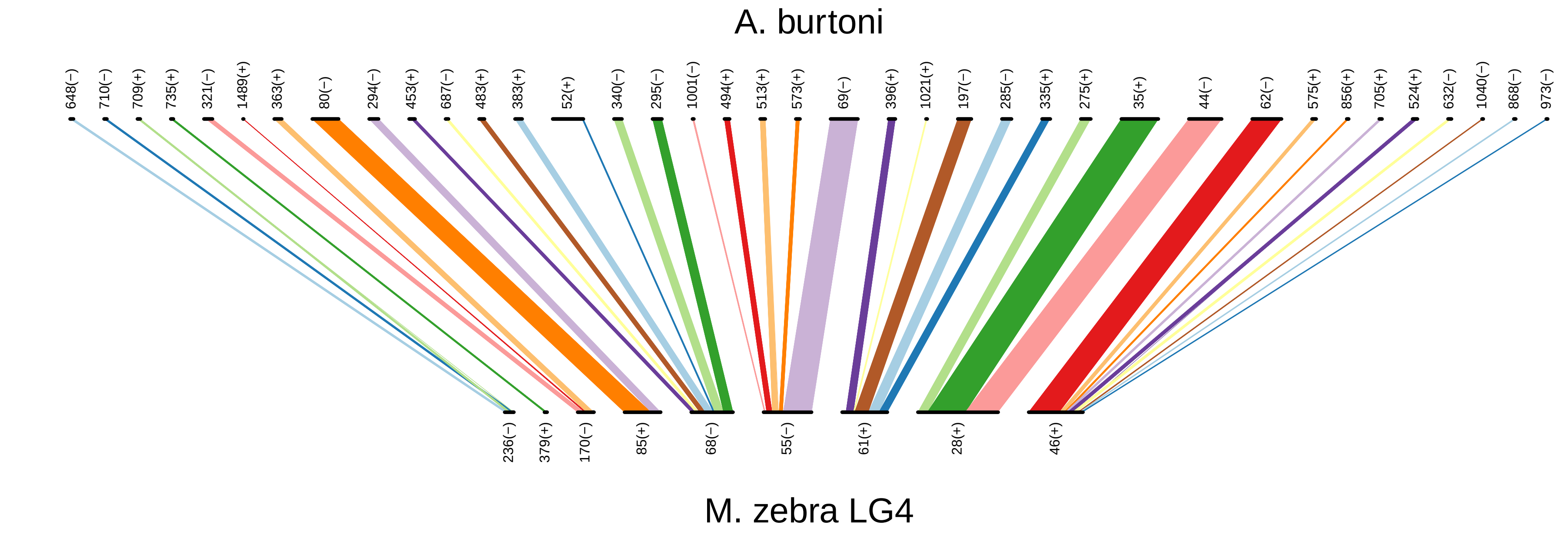

A. burtoni
648(−)
710(−)
709(+)
735(+)
321(−)
1489(+)
363(+)
80(−)
294(−)
453(+)
687(−)
483(+)
383(+)
52(+)
340(−)
295(−)
1001(−)
494(+)
513(+)
573(+)
69(−)
396(+)
1021(+)
197(−)
285(−)
335(+)
275(+)
35(+)
44(−)
62(−)
575(+)
856(+)
705(+)
524(+)
632(−)
1040(−)
868(−)
973(−)
236(−)
379(+)
170(−)
85(+)
68(−)
55(−)
61(+)
28(+)
46(+)
M. zebra LG4

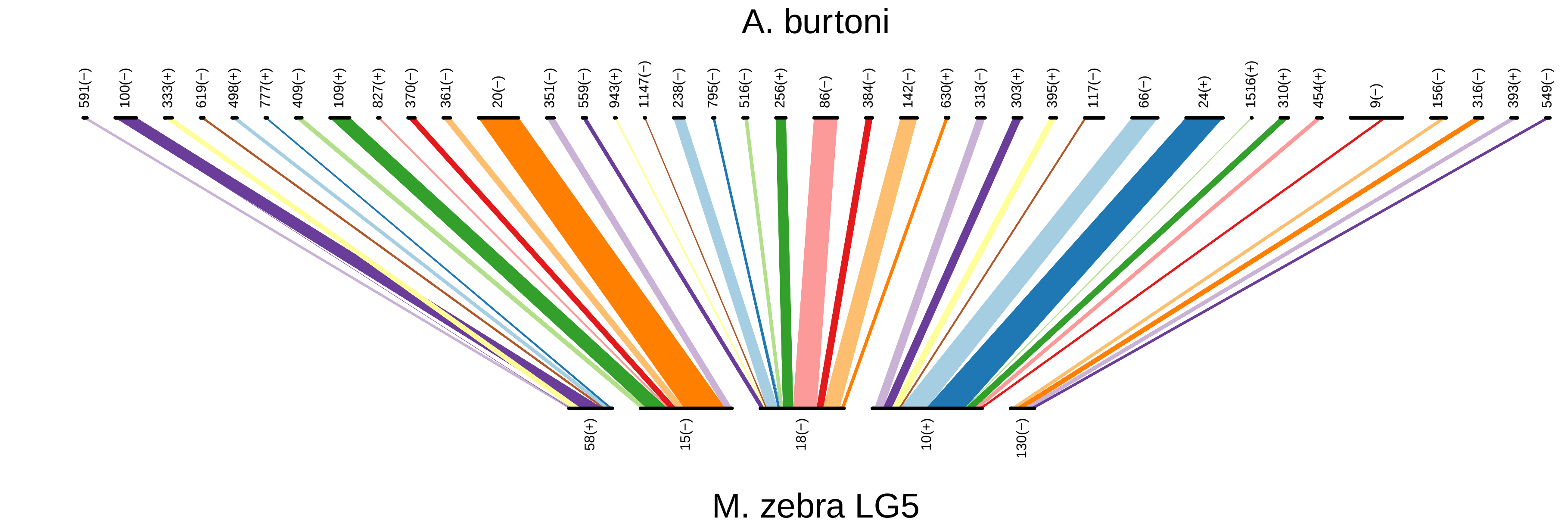
A. burtoni
591(−)
100(−)
333(+)
619(−)
498(+)
777(+)
409(−)
109(+)
827(+)
370(−)
361(−)
20(−)
351(−)
559(−)
943(+)
1147(−)
238(−)
795(−)
516(−)
256(+)
86(−)
384(−)
142(−)
630(+)
313(−)
303(+)
395(+)
117(−)
66(−)
24(+)
1516(+)
310(+)
454(+)
9(−)
156(−)
316(−)
393(+)
549(−)
58(+)
15(−)
18(−)
10(+)
130(−)
M. zebra LG5

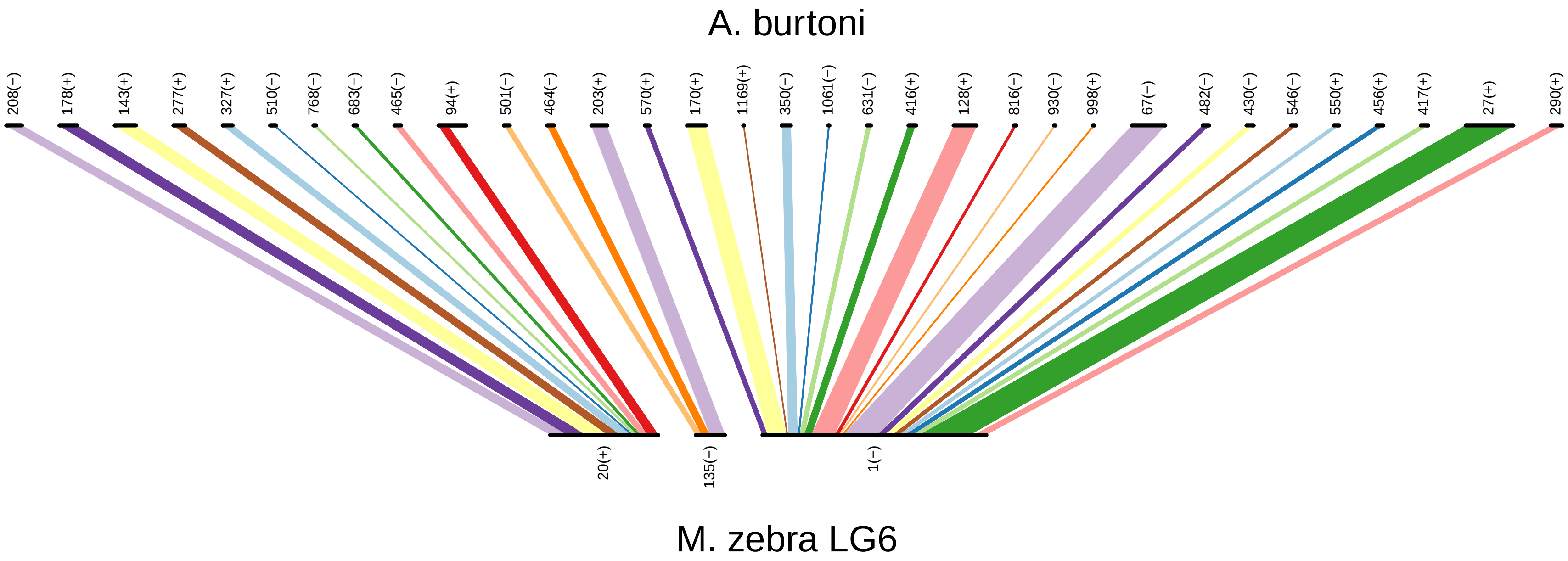

A. burtoni
208(−)
178(+)
143(+)
277(+)
327(+)
510(−)
768(−)
683(−)
465(−)
94(+)
501(−)
464(−)
203(+)
570(+)
170(+)
1169(+)
350(−)
1061(−)
631(−)
416(+)
128(+)
816(−)
930(−)
998(+)
67(−)
482(−)
430(−)
546(−)
550(+)
456(+)
417(+)
27(+)
290(+)
20(+)
135(−)
1(−)
M. zebra LG6

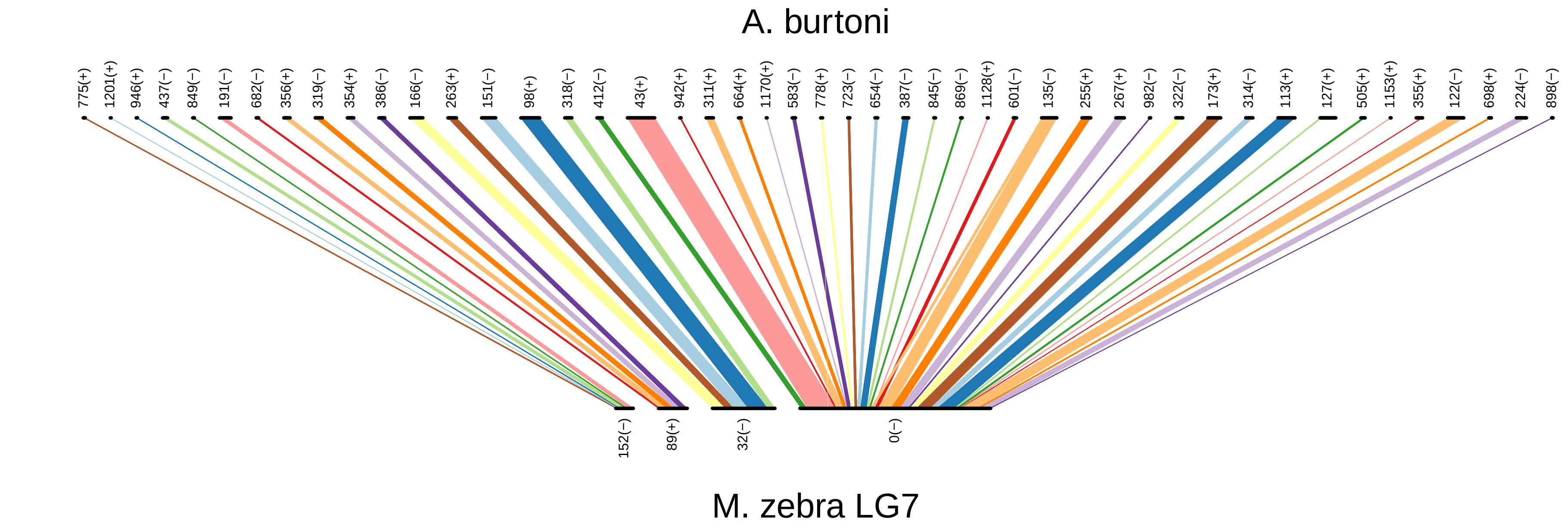

A. burtoni
775(+)
1201(+)
946(+)
437(−)
849(−)
191(−)
682(−)
356(+)
319(−)
354(+)
386(−)
166(−)
263(+)
151(−)
98(+)
318(−)
412(−)
43(+)
942(+)
311(+)
664(+)
1170(+)
583(−)
778(+)
723(−)
654(−)
387(−)
845(−)
869(−)
1128(+)
601(−)
135(−)
255(+)
267(+)
982(−)
322(−)
173(+)
314(−)
113(+)
127(+)
505(+)
1153(+)
355(+)
122(−)
698(+)
224(−)
898(−)
152(−)
89(+)
32(−)
0(−)
M. zebra LG7

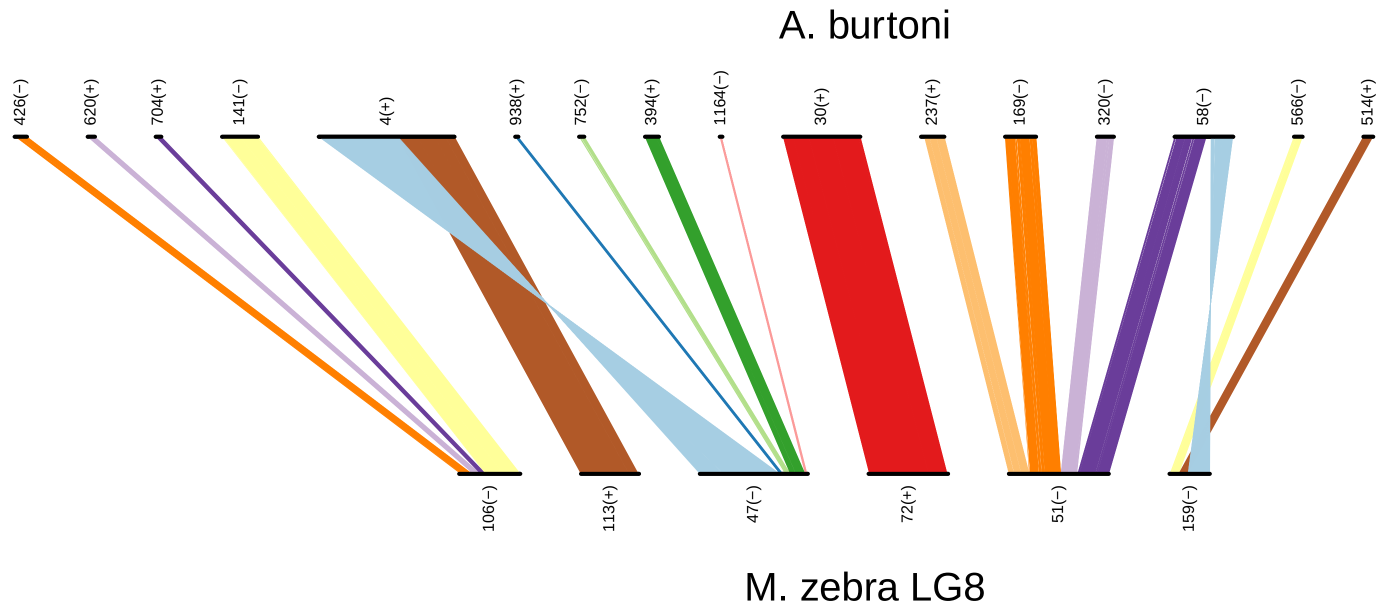

A. burtoni
426(−)
620(+)
704(+)
141(−)
4(+)
938(+)
752(−)
394(+)
1164(−)
30(+)
237(+)
169(−)
320(−)
58(−)
566(−)
514(+)
106(−)
113(+)
47(−)
72(+)
51(−)
159(−)
M. zebra LG8

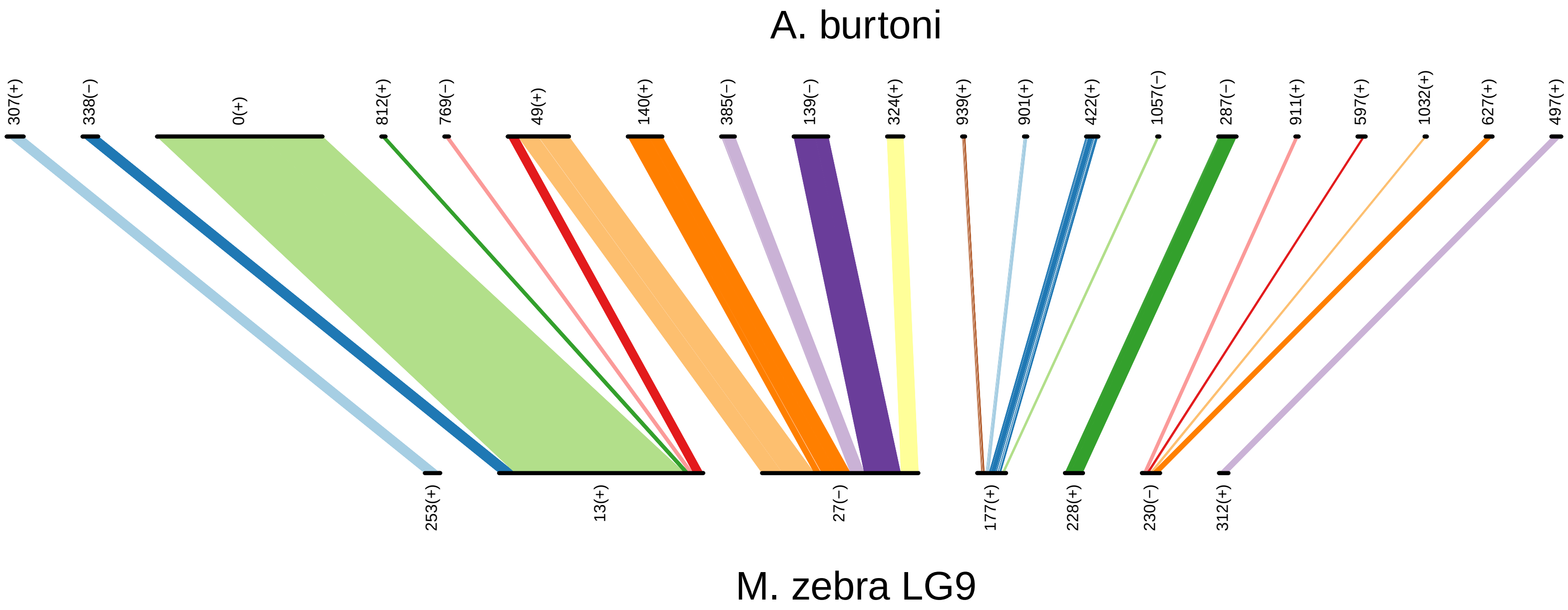

A. burtoni
307(+)
338(−)
0(+)
812(+)
769(−)
49(+)
140(+)
385(−)
139(−)
324(+)
939(+)
901(+)
422(+)
1057(−)
287(−)
911(+)
597(+)
1032(+)
627(+)
497(+)
253(+)
13(+)
27(−)
177(+)
228(+)
230(−)
312(+)
M. zebra LG9

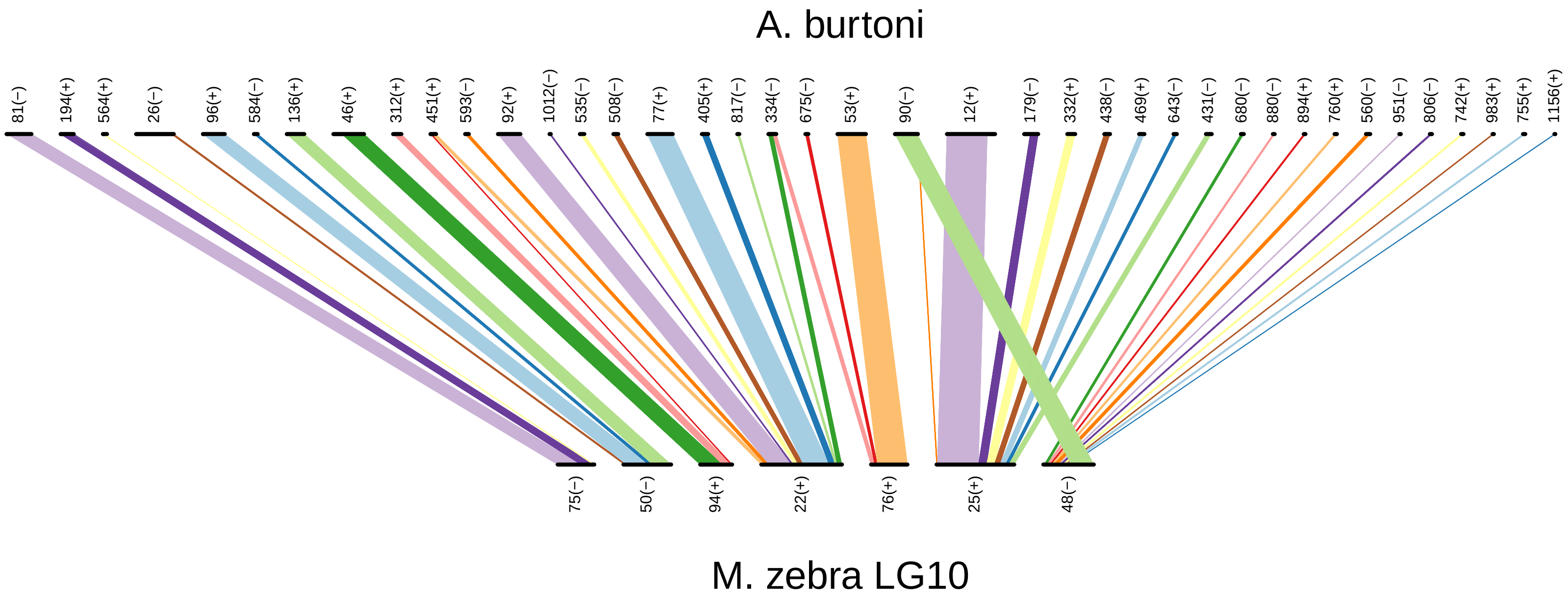

A. burtoni
81(−)
194(+)
564(+)
26(−)
96(+)
584(−)
136(+)
46(+)
312(+)
451(+)
593(−)
92(+)
1012(−)
535(−)
508(−)
77(+)
405(+)
817(−)
334(−)
675(−)
53(+)
90(−)
12(+)
179(−)
332(+)
438(−)
469(+)
643(−)
431(−)
680(−)
880(−)
894(+)
760(+)
560(−)
951(−)
806(−)
742(+)
983(+)
755(+)
1156(+)
75(−)
50(−)
94(+)
22(+)
76(+)
25(+)
48(−)
M. zebra LG10

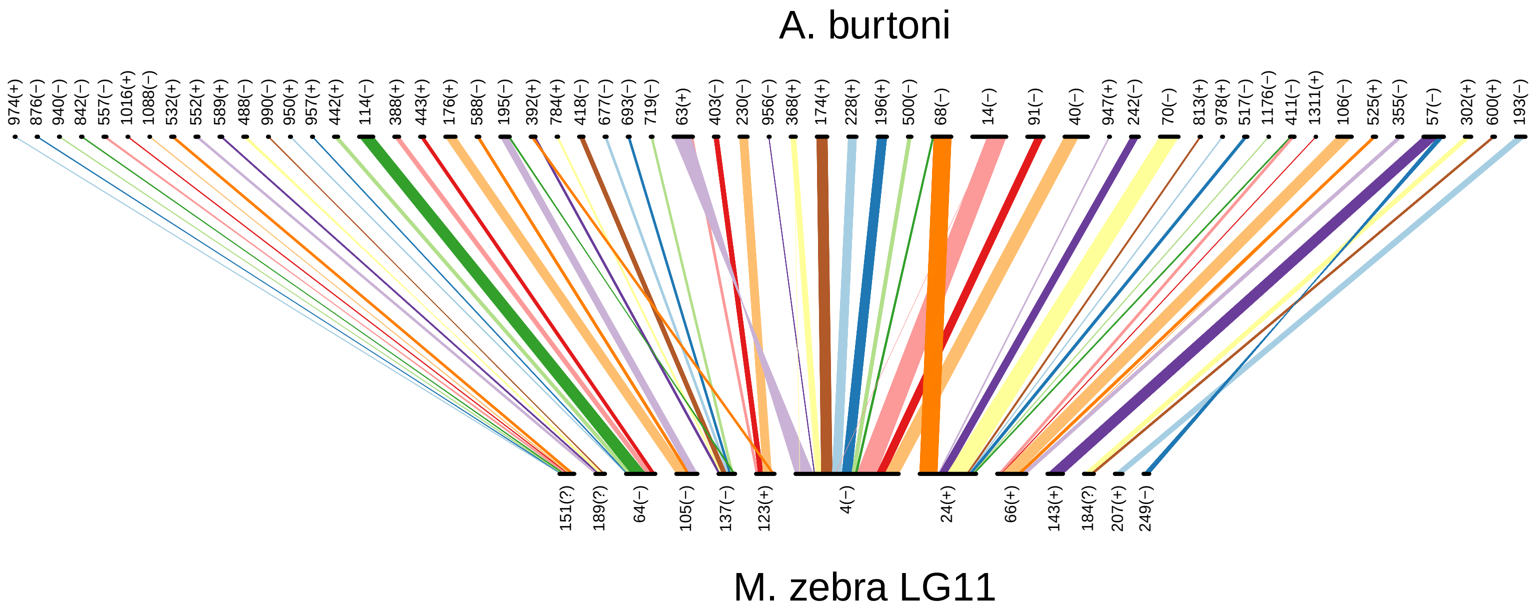
A. burtoni
974(+)
876(−)
940(−)
842(−)
557(−)
1016(+)
1088(−)
532(+)
552(+)
589(+)
488(−)
990(−)
950(+)
957(+)
442(+)
114(−)
388(+)
443(+)
176(+)
588(−)
195(−)
392(+)
784(+)
418(−)
677(−)
693(−)
719(−)
63(+)
403(−)
230(−)
956(−)
368(+)
174(+)
228(+)
196(+)
500(−)
68(−)
14(−)
91(−)
40(−)
947(+)
242(−)
70(−)
813(+)
978(+)
517(−)
1176(−)
411(−)
1311(+)
106(−)
525(+)
355(−)
57(−)
302(+)
600(+)
193(−)
151(?)
189(?)
64(−)
105(−)
137(−)
123(+)
4(−)
24(+)
66(+)
143(+)
184(?)
207(+)
249(−)
M. zebra LG11

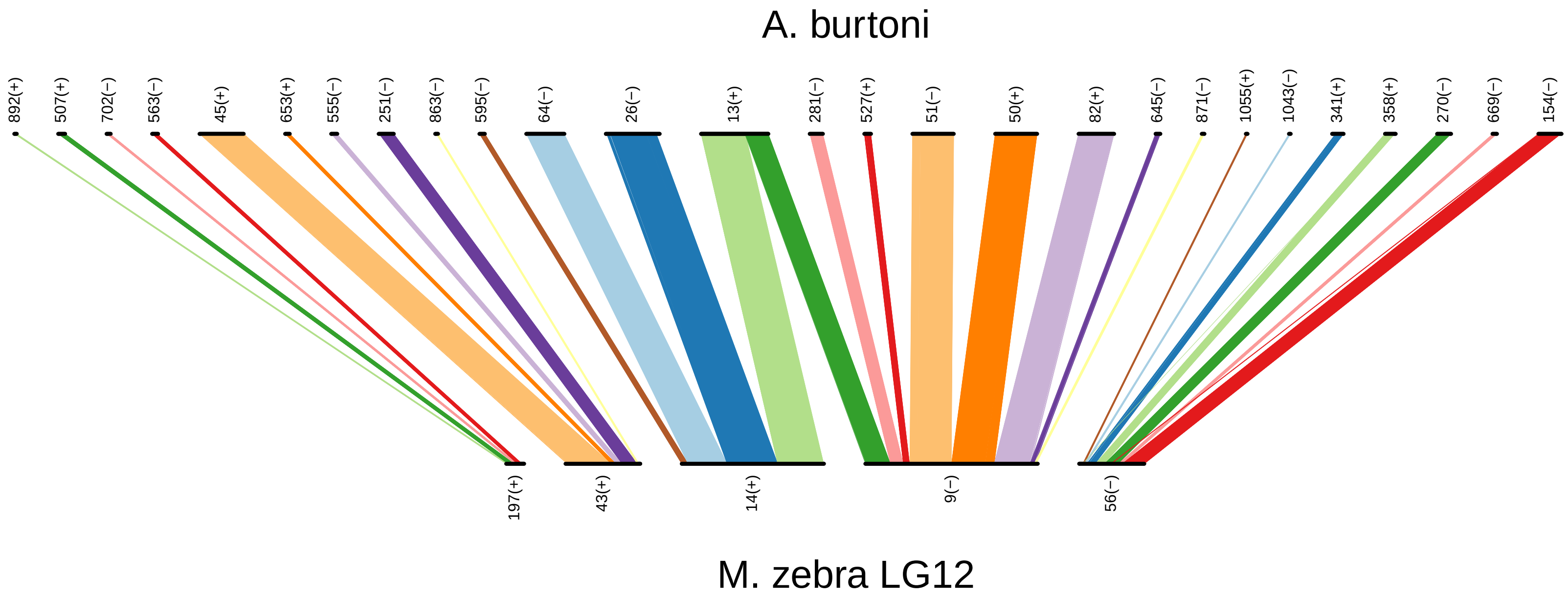

A. burtoni
892(+)
507(+)
702(−)
563(−)
45(+)
653(+)
555(−)
251(−)
863(−)
595(−)
64(−)
26(−)
13(+)
281(−)
527(+)
51(−)
50(+)
82(+)
645(−)
871(−)
1055(+)
1043(−)
341(+)
358(+)
270(−)
669(−)
154(−)
197(+)
43(+)
14(+)
9(−)
56(−)
M. zebra LG12

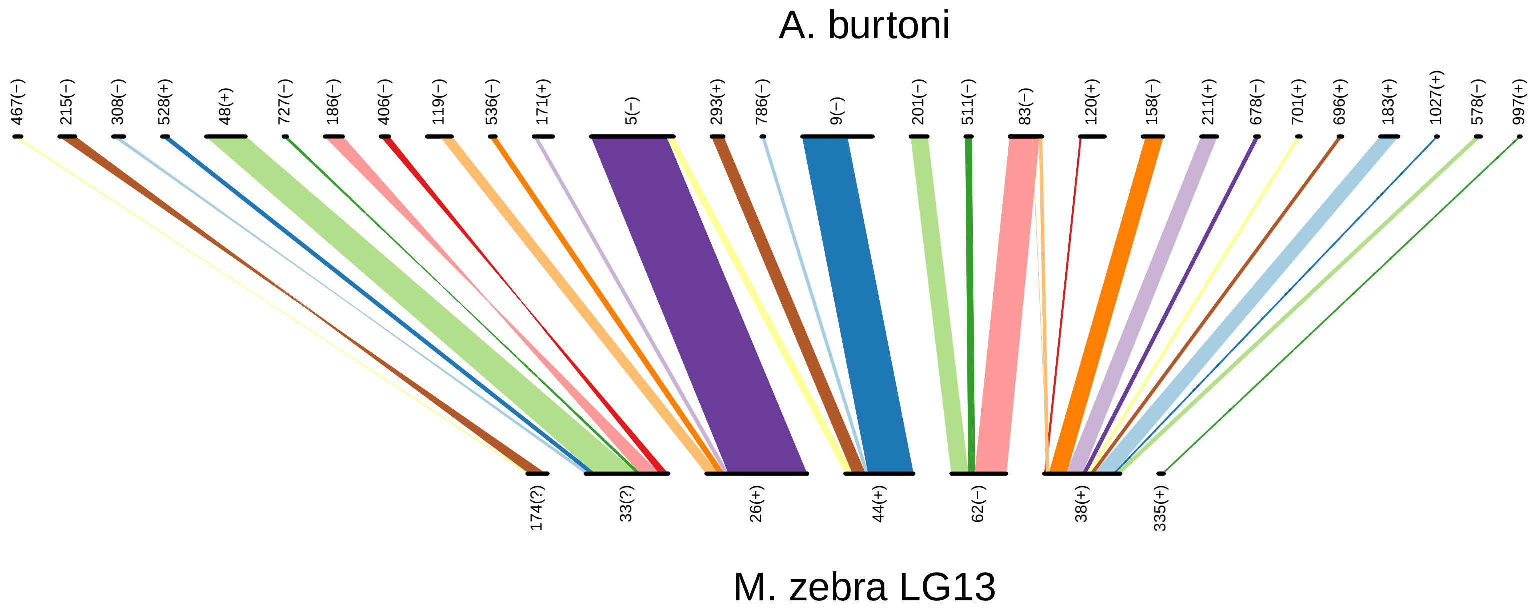
A. burtoni
467(−)
215(−)
308(−)
528(+)
48(+)
727(−)
186(−)
406(−)
119(−)
536(−)
171(+)
5(−)
293(+)
786(−)
9(−)
201(−)
511(−)
83(−)
120(+)
158(−)
211(+)
678(−)
701(+)
696(+)
183(+)
1027(+)
578(−)
997(+)
174(?)
33(?)
26(+)
44(+)
62(−)
38(+)
335(+)
M. zebra LG13

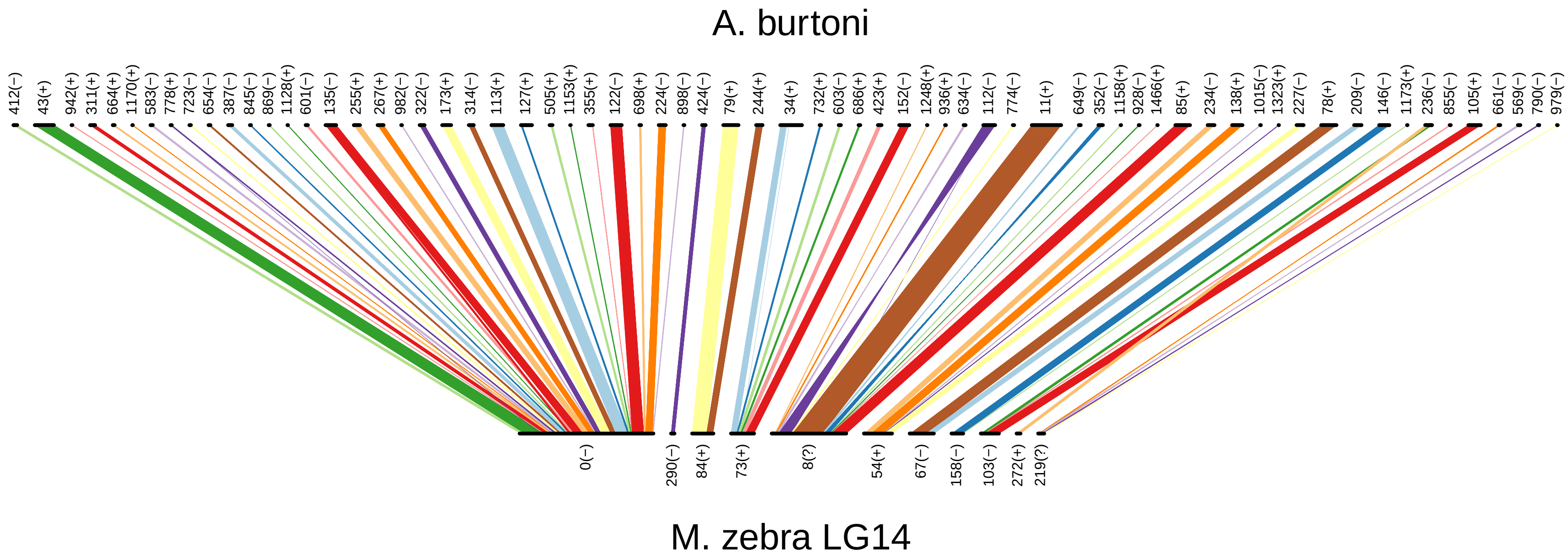

A. burtoni
412(−)
43(+)
942(+)
311(+)
664(+)
1170(+)
583(−)
778(+)
723(−)
654(−)
387(−)
845(−)
869(−)
1128(+)
601(−)
135(−)
255(+)
267(+)
982(−)
322(−)
173(+)
314(−)
113(+)
127(+)
505(+)
1153(+)
355(+)
122(−)
698(+)
224(−)
898(−)
424(−)
79(+)
244(+)
34(+)
732(+)
603(−)
686(+)
423(+)
152(−)
1248(+)
936(+)
634(−)
112(−)
774(−)
11(+)
649(−)
352(−)
1158(+)
928(−)
1466(+)
85(+)
234(−)
138(+)
1015(−)
1323(+)
227(−)
78(+)
209(−)
146(−)
1173(+)
236(−)
855(−)
105(+)
661(−)
569(−)
790(−)
979(−)
0(−)
290(−)
84(+)
73(+)
8(?)
54(+)
67(−)
158(−)
103(−)
272(+)
219(?)
M. zebra LG14

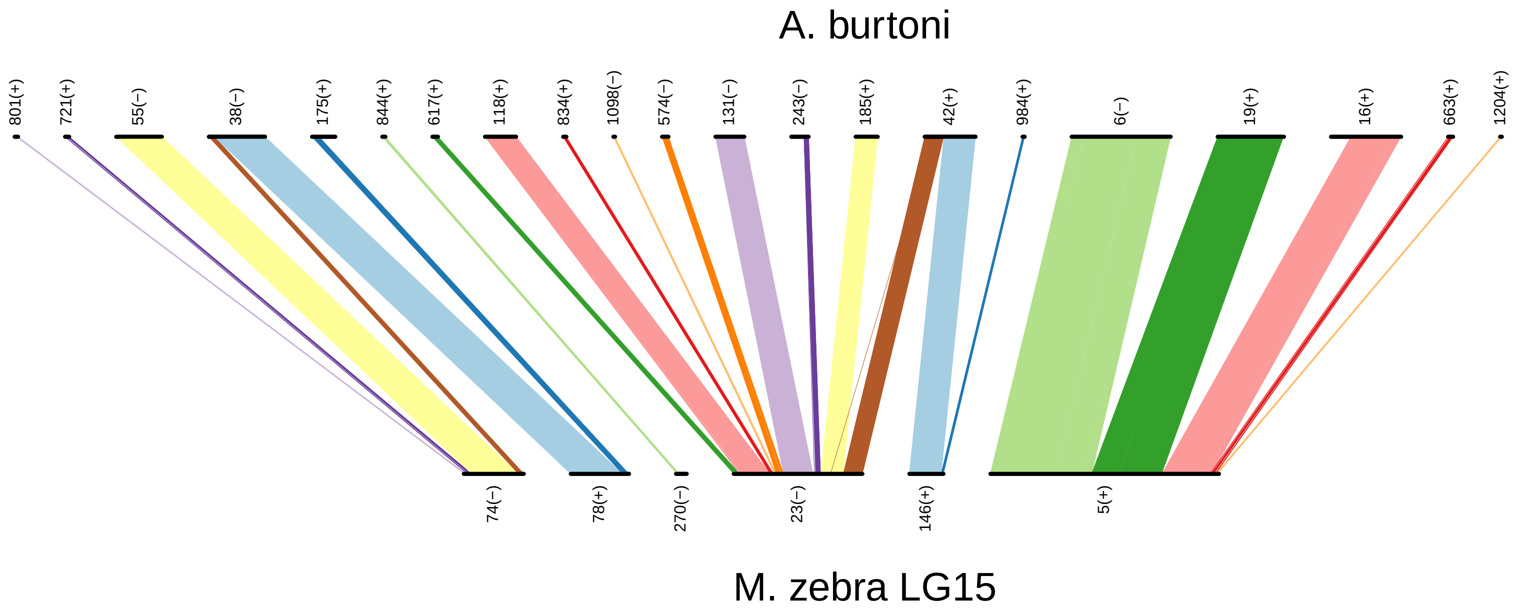
A. burtoni
801(+)
721(+)
55(−)
38(−)
175(+)
844(+)
617(+)
118(+)
834(+)
1098(−)
574(−)
131(−)
243(−)
185(+)
42(+)
984(+)
6(−)
19(+)
16(+)
663(+)
1204(+)
74(−)
78(+)
270(−)
23(−)
146(+)
5(+)
M. zebra LG15

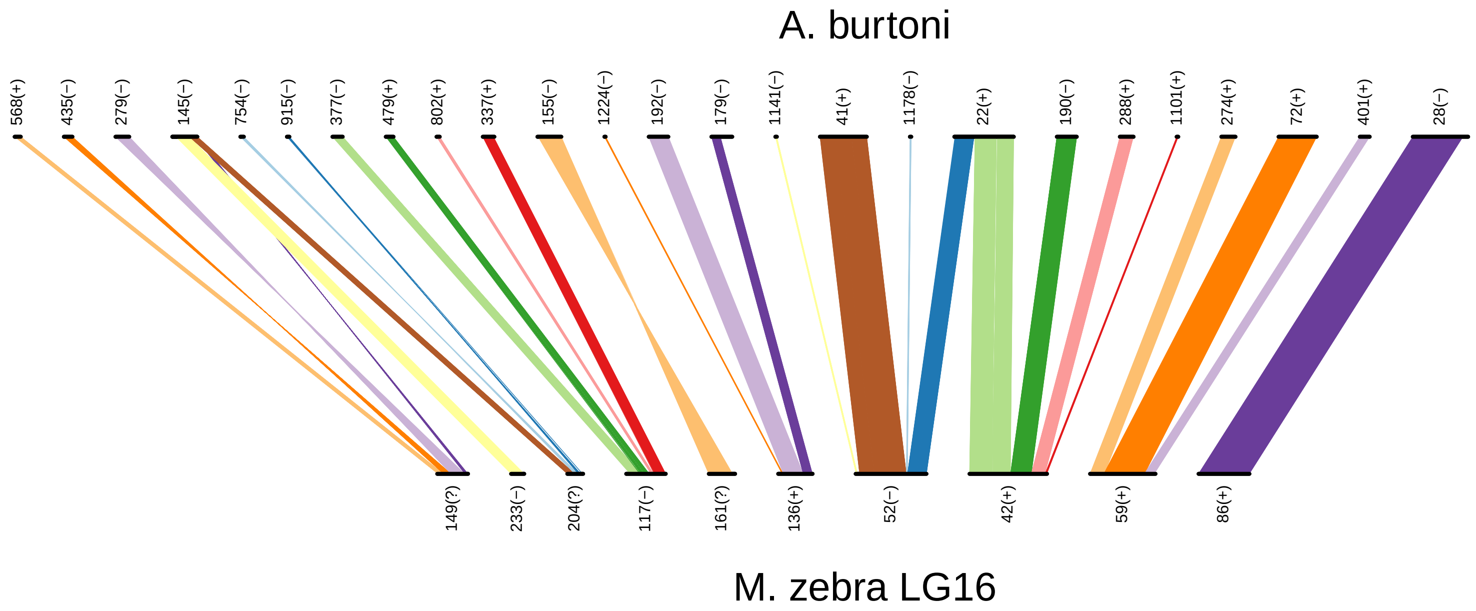
A. burtoni
568(+)
435(−)
279(−)
145(−)
754(−)
915(−)
377(−)
479(+)
802(+)
337(+)
155(−)
1224(−)
192(−)
179(−)
1141(−)
41(+)
1178(−)
22(+)
190(−)
288(+)
1101(+)
274(+)
72(+)
401(+)
28(−)
149(?)
233(−)
204(?)
117(−)
161(?)
136(+)
52(−)
42(+)
59(+)
86(+)
M. zebra LG16

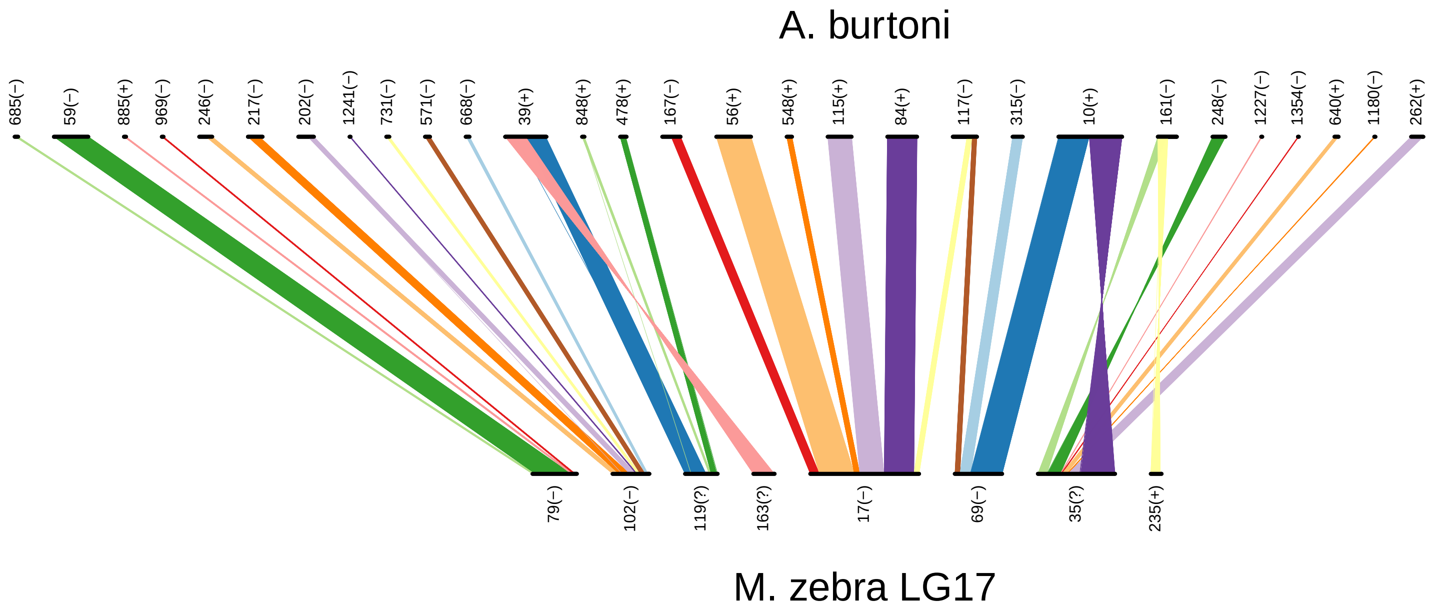
A. burtoni
685(−)
59(−)
885(+)
969(−)
246(−)
217(−)
202(−)
1241(−)
731(−)
571(−)
668(−)
39(+)
848(+)
478(+)
167(−)
56(+)
548(+)
115(+)
84(+)
117(−)
315(−)
10(+)
161(−)
248(−)
1227(−)
1354(−)
640(+)
1180(−)
262(+)
79(−)
102(−)
119(?)
163(?)
17(−)
69(−)
35(?)
235(+)
M. zebra LG17

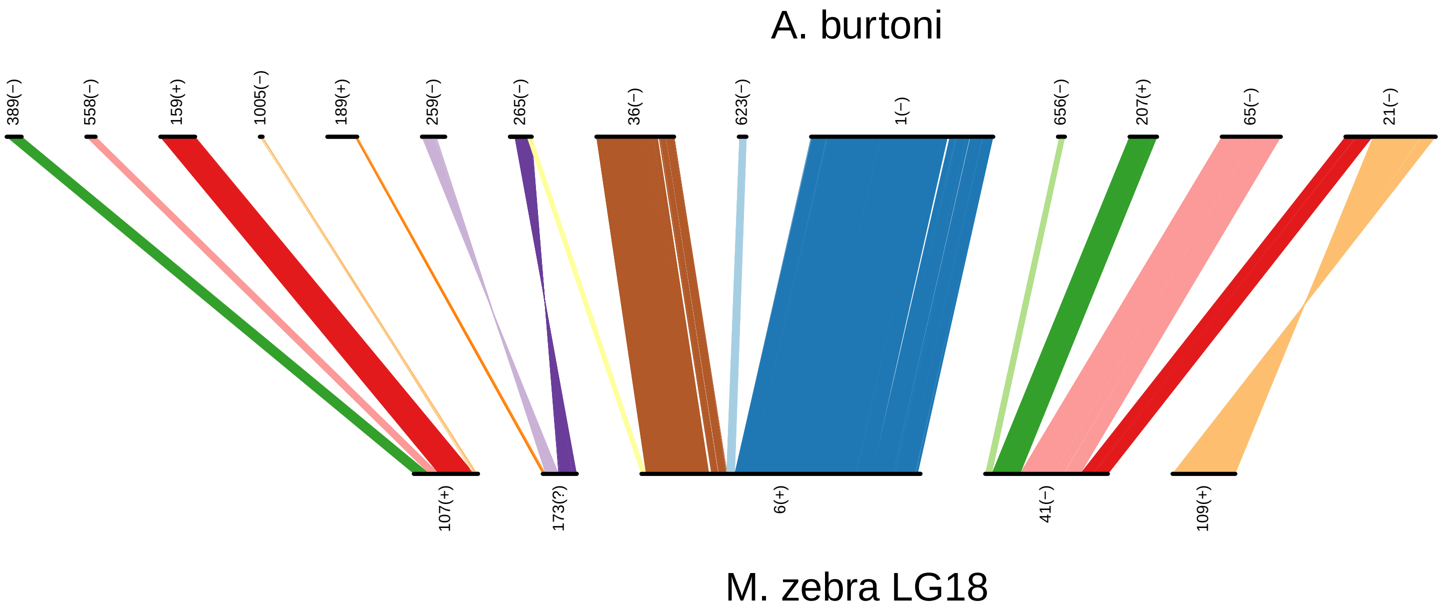
A. burtoni
389(−)
558(−)
159(+)
1005(−)
189(+)
259(−)
265(−)
36(−)
623(−)
1(−)
656(−)
207(+)
65(−)
21(−)
107(+)
173(?)
6(+)
41(−)
109(+)
M. zebra LG18

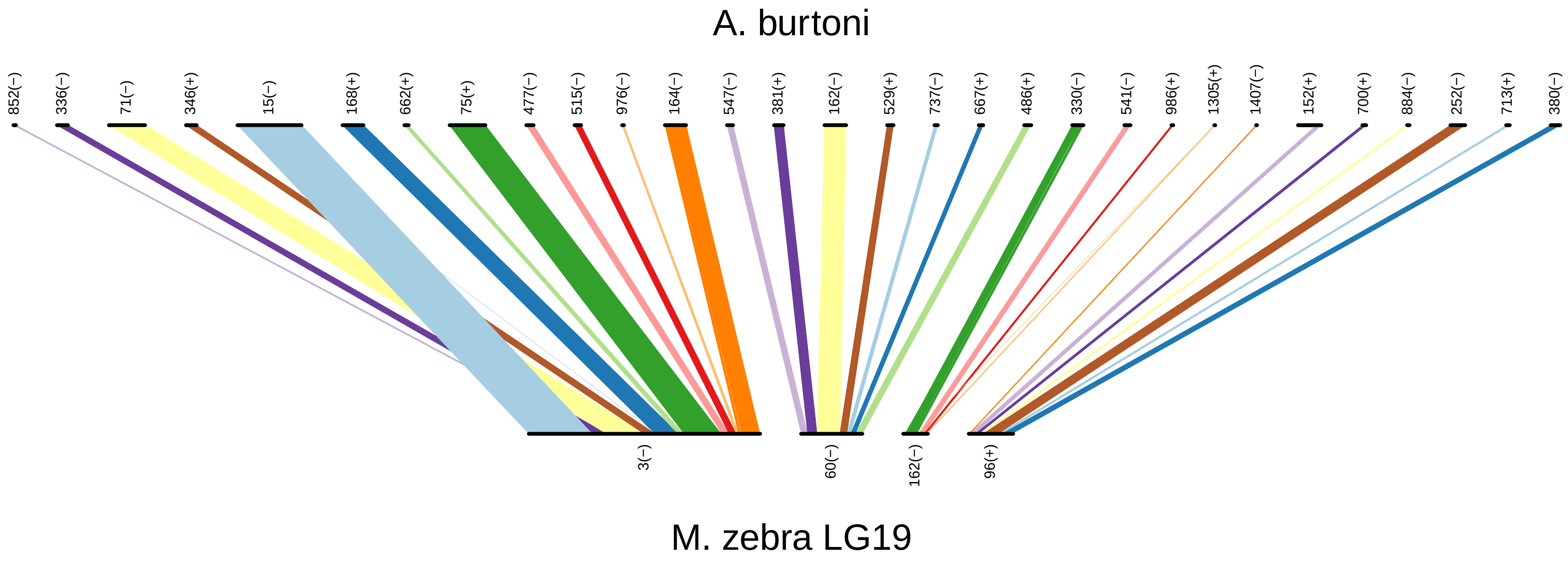

A. burtoni
852(−)
336(−)
71(−)
346(+)
15(−)
168(+)
662(+)
75(+)
477(−)
515(−)
976(−)
164(−)
547(−)
381(+)
162(−)
529(+)
737(−)
667(+)
486(+)
330(−)
541(−)
986(+)
1305(+)
1407(−)
152(+)
700(+)
884(−)
252(−)
713(+)
380(−)
3(−)
60(−)
162(−)
96(+)
M. zebra LG19

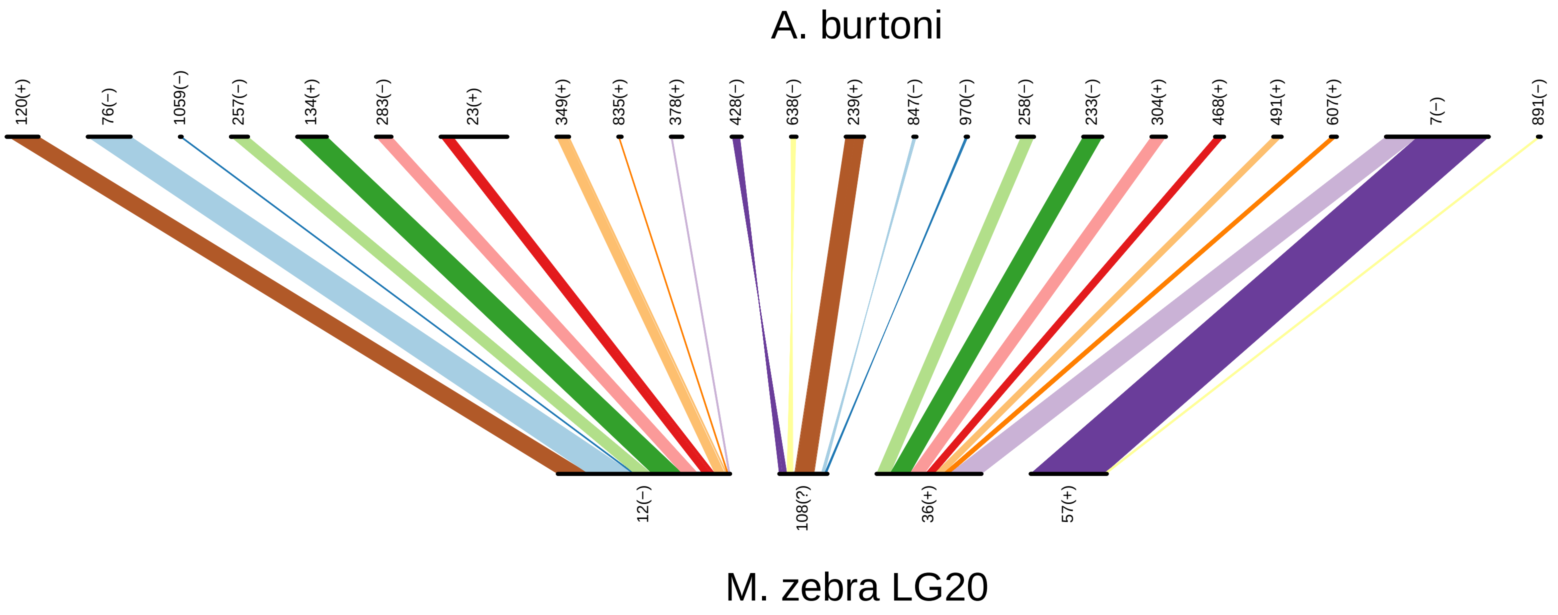
A. burtoni
120(+)
76(−)
1059(−)
257(−)
134(+)
283(−)
23(+)
349(+)
835(+)
378(+)
428(−)
638(−)
239(+)
847(−)
970(−)
258(−)
233(−)
304(+)
468(+)
491(+)
607(+)
7(−)
891(−)
12(−)
108(?)
36(+)
57(+)
M. zebra LG20

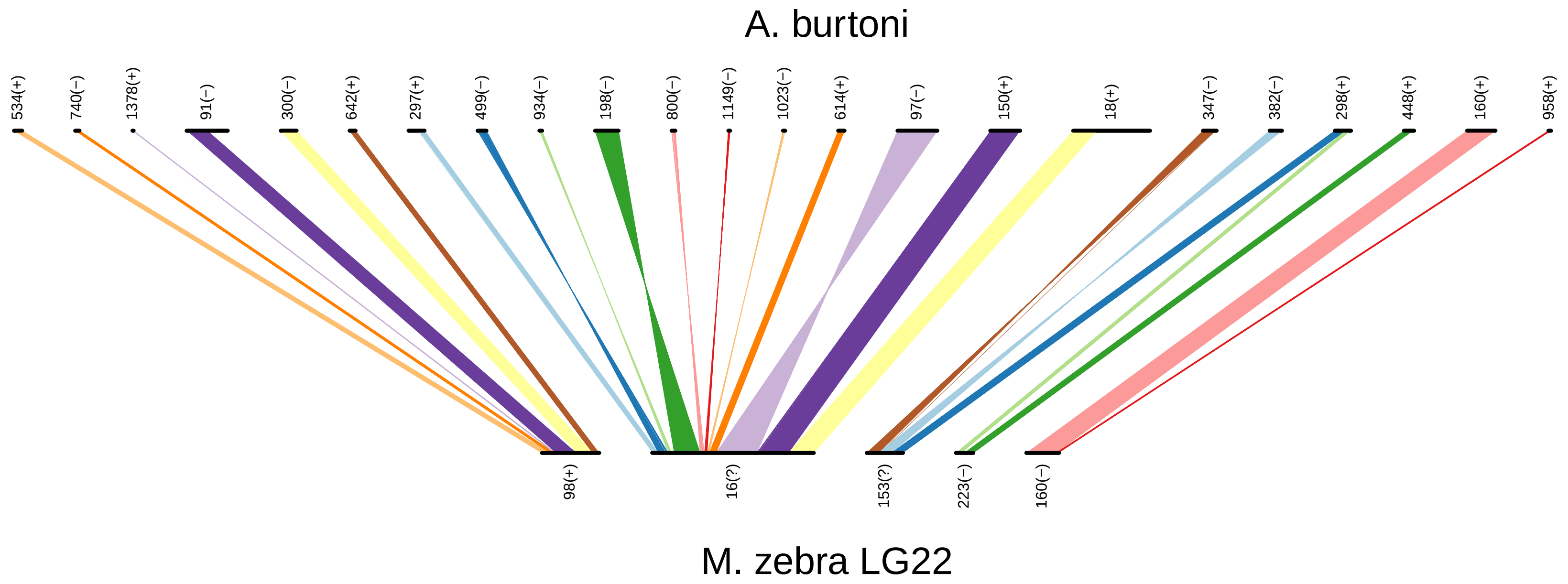
A. burtoni
534(+)
740(−)
1378(+)
91(−)
300(−)
642(+)
297(+)
499(−)
934(−)
198(−)
800(−)
1149(−)
1023(−)
614(+)
97(−)
150(+)
18(+)
347(−)
382(−)
298(+)
448(+)
160(+)
958(+)
98(+)
16(?)
153(?)
223(−)
160(−)
M. zebra LG22

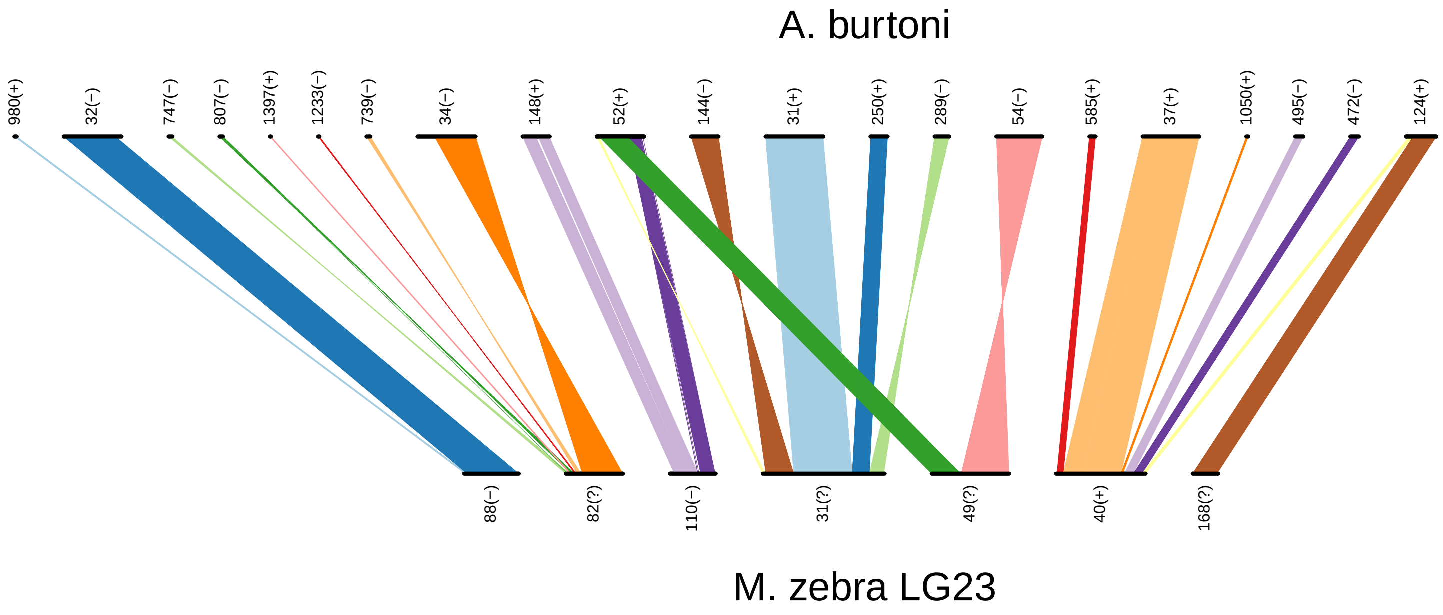
A. burtoni
980(+)
32(−)
747(−)
807(−)
1397(+)
1233(−)
739(−)
34(−)
148(+)
52(+)
144(−)
31(+)
250(+)
289(−)
54(−)
585(+)
37(+)
1050(+)
495(−)
472(−)
124(+)
88(−)
82(?)
110(−)
31(?)
49(?)
40(+)
168(?)
M. zebra LG23
